# Supplementary material for: Characterization of Bunch Compactness in a Diverse Collection of Vitis vinifera L. Genotypes Enriched in Table Grape Cultivars Reveals New Candidate Genes Associated with Berry Number
Source: Plants (Basel). 2025 Apr 26;14(9):1308. doi: 10.3390/plants14091308 (PMC12073236; doi:10.3390/plants14091308)
Supplement: Supplementary file 1 [file plants-14-01308-s001.zip › Captions - Supp Material.pdf]

Supplementary

Figures

Figure S1. Contributions of variables to the first and second principal components of multivariate analysis over two seasons.

Figure S2. Histogram for compactness indexes (as proposed in Tello & Ibañez 2015) during the first season.

Figure S3. Multivariant analysis of quantitative traits describing bunch compactness considering values from both ground measurements and automated phenotyping.

Figure S4. Discriminant analysis of principal components using the genetic data of informative SNPs for the studied collection. (A) Scatter plot using the first and second linear discriminant (B) Bayesian Information Criterion to estimate the most probable true value of number of groups (C) Cumulative variance graph ordered by principal components

Figure S5. Identification of SNPs associated with Compactness Index – 3.

Figure S6. Venn diagram showing the common SNPs between all the unique sites detected among different seasons.

Figure S7. Idiogram of the significant SNPs associated with their corresponding trait over two seasons.

Figure S8. Enrichment analysis of the GO terms in the list of genes associated with the 92 common SNPs found in both seasons. A total of 64 genes were successfully mapped.

Tables

Table S1. Plant material used for the characterization of traits determining bunch compactness. A collection of cultivars with high genetic diversity due to their different provenances and reported utilization was considered for the study of traits involved in the compactness phenotype. There is a particular enrichment on modern table grape material which was not subjected to standard management practices regarding usage of plant growth regulator for thinning and berry enlargement since our focus was to observe the phenotype under a basal condition. Classification and provenance are those reported in the *Vitis* International Variety Catalogue (*V*IVC).

Table S2. Descriptive statistics for quantitative indexes describing BC. The compactness indexes proposed by Tello & Ibañez (2015) were calculated for our dataset across two seasons. Descriptive statistics are shown for a total of 768 and 622 observations for the first and second seasons, respectively.

Table S3. Full list of the total detected signals with significant association across all traits and seasons studied.

Table S4. Summary of the predicted effects of the alternative alleles on the closest annotated genes. The predicted effect based on the alternative allele is reported. Categories

are those reported by Snpeff (Cingolani et al., 2012). Some sites could have multiple predicted effects but only one of them was considered.

Files

Supplementary File S1. Quality control of the variants yield by the Genotyping-by-Sequencing analysis before filtering.

Supplementary File S2. Quality control of the variants yield by the Genotyping-by-Sequencing analysis after filtering.
