# Supplementary material for: Characterization of Bunch Compactness in a Diverse Collection of Vitis vinifera L. Genotypes Enriched in Table Grape Cultivars Reveals New Candidate Genes Associated with Berry Number
Source: Plants (Basel). 2025 Apr 26;14(9):1308. doi: 10.3390/plants14091308 (PMC12073236; doi:10.3390/plants14091308)
Supplement: Supplementary file 1 [file plants-14-01308-s001.zip › Supplementary File S1 - QC inspection.pdf]

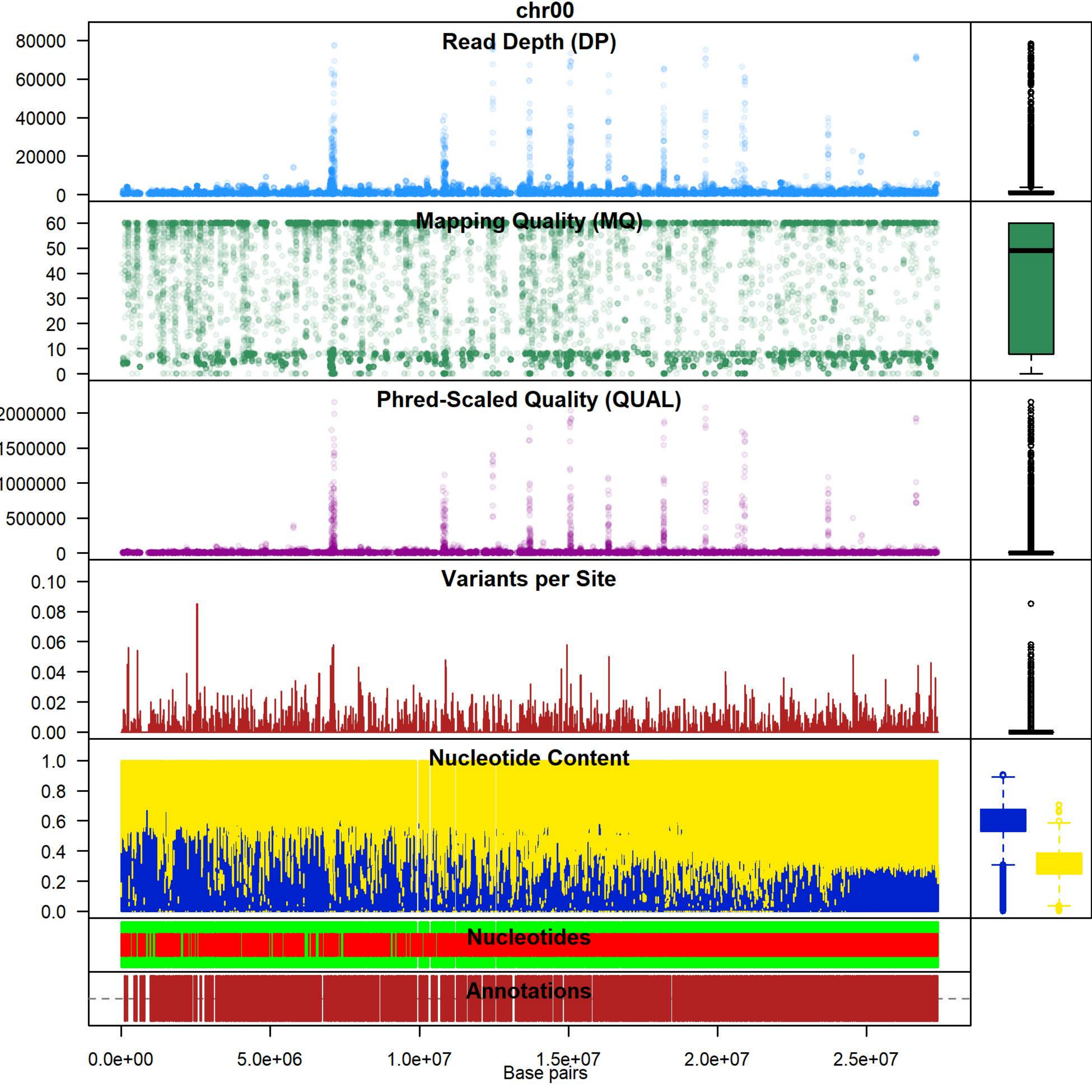
chr00
Read Depth (DP)
80000
60000
40000
20000
0
Mapping Quality (MQ)
60
50
40
30
20
10
0
Phred-Scaled Quality (QUAL)
2000000
1500000
1000000
500000
0
Variants per Site
0.10
0.08
0.06
0.04
0.02
0.00
Nucleotide Content
1.0
0.8
0.6
0.4
0.2
0.0
Nucleotides
Annotations
0.0e+00
5.0e+06
1.0e+07
1.5e+07
2.0e+07
2.5e+07
Base pairs

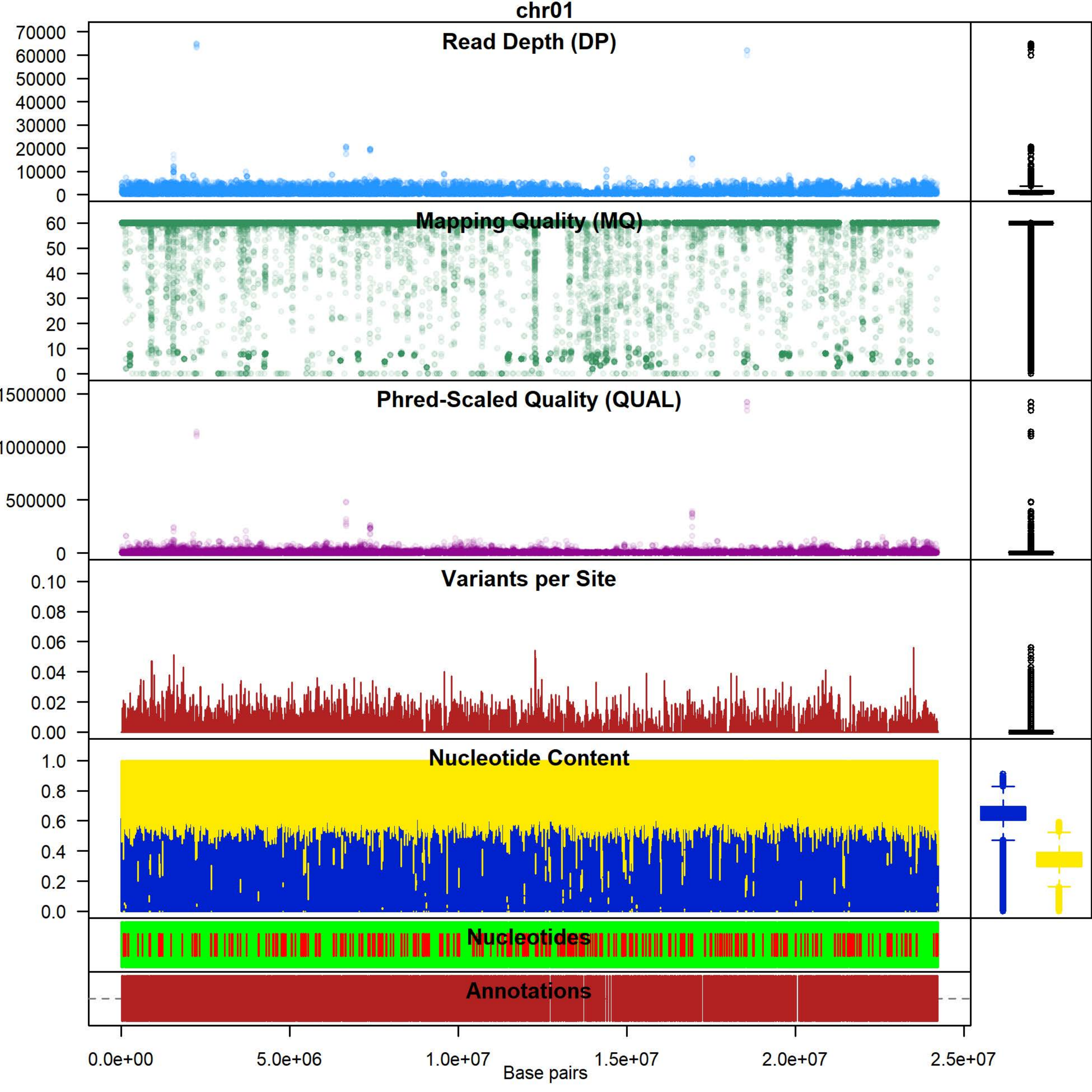

chr01
Read Depth (DP)
70000
60000
50000
40000
30000
20000
10000
0
Mapping Quality (MQ)
60
50
40
30
20
10
0
Phred-Scaled Quality (QUAL)
1500000
1000000
500000
0
Variants per Site
0.10
0.08
0.06
0.04
0.02
0.00
Nucleotide Content
1.0
0.8
0.6
0.4
0.2
0.0
Nucleotides
Annotations
0.0e+00
5.0e+06
1.0e+07
1.5e+07
2.0e+07
2.5e+07
Base pairs

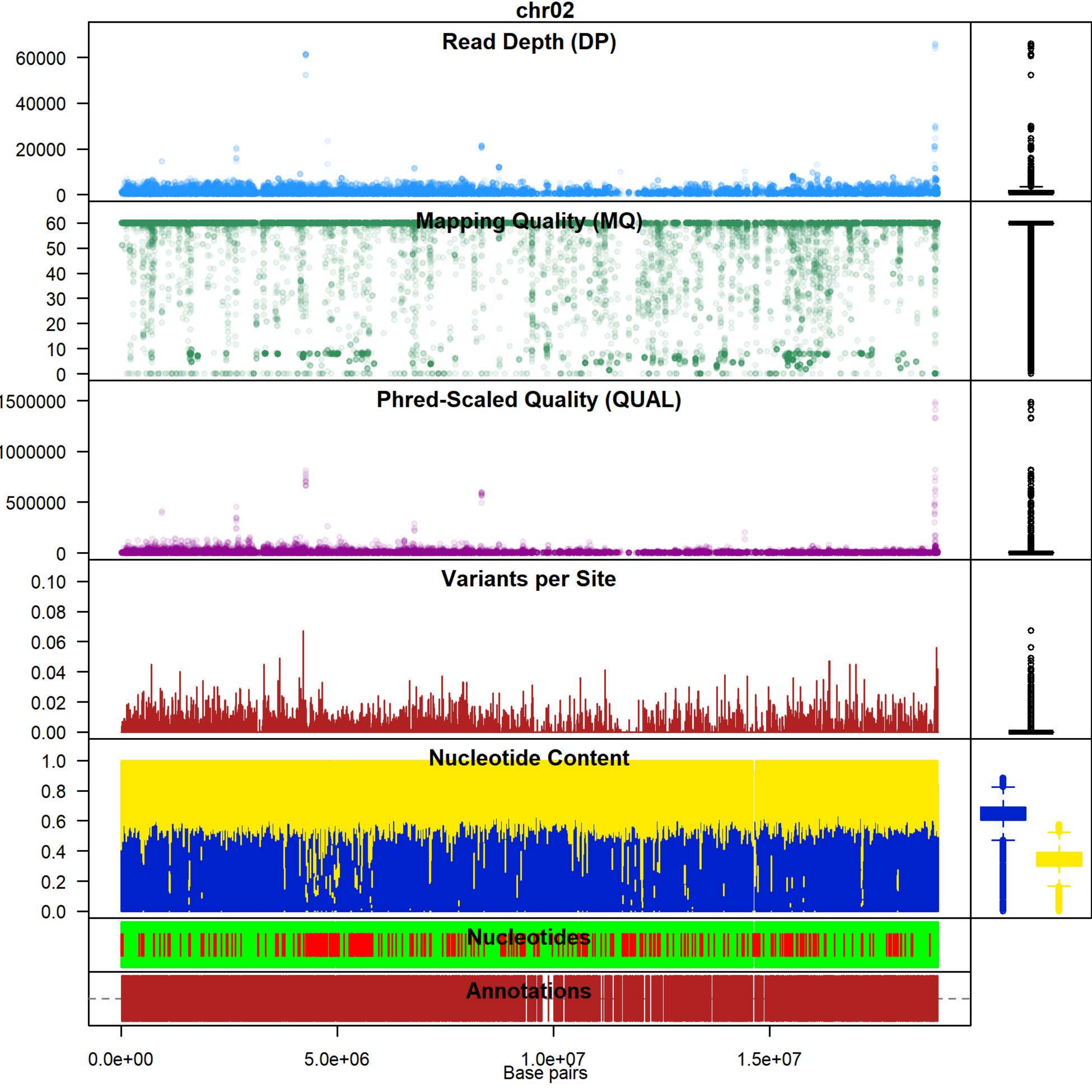

chr02
Read Depth (DP)
60000
40000
20000
0
Mapping Quality (MQ)
60
50
40
30
20
10
0
Phred-Scaled Quality (QUAL)
1500000
1000000
500000
0
Variants per Site
0.10
0.08
0.06
0.04
0.02
0.00
Nucleotide Content
1.0
0.8
0.6
0.4
0.2
0.0
Nucleotides
Annotations
0.0e+00
5.0e+06
1.0e+07
1.5e+07
Base pairs

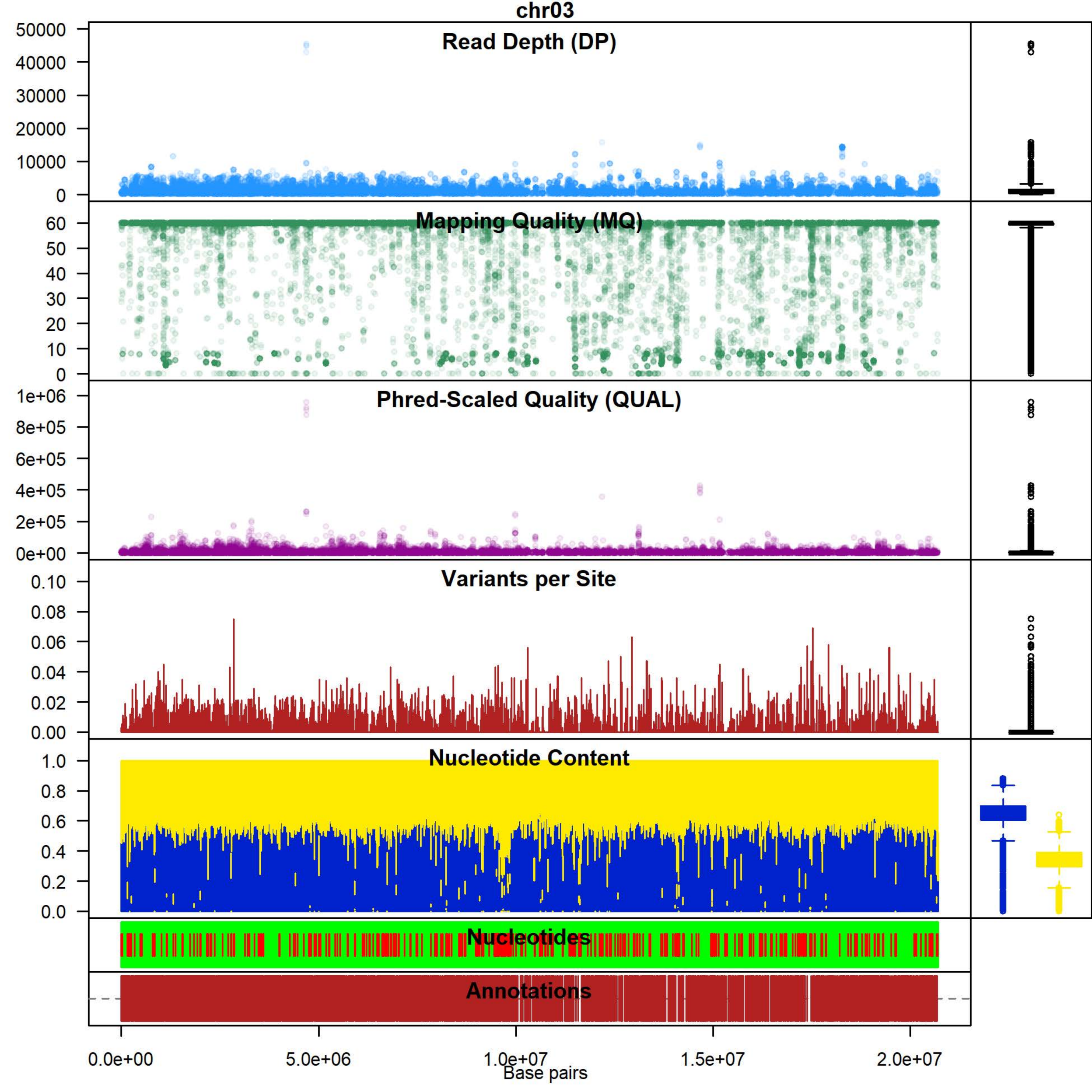

chr03
Read Depth (DP)
Mapping Quality (MQ)
Phred-Scaled Quality (QUAL)
Variants per Site
Nucleotide Content
Nucleotides
Annotations
0.0e+00
5.0e+06
1.0e+07
1.5e+07
2.0e+07
Base pairs

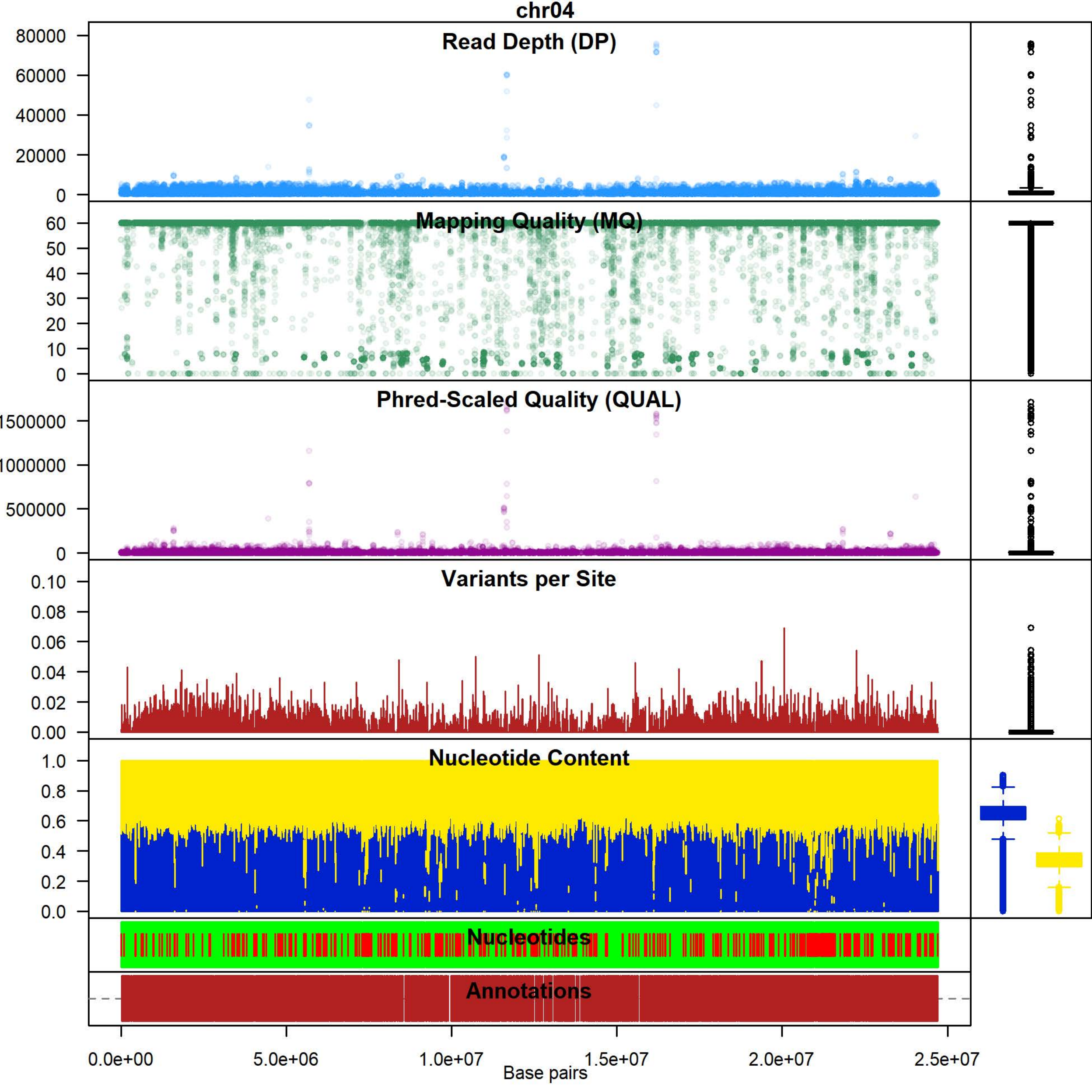
chr04
Read Depth (DP)
Mapping Quality (MQ)
Phred-Scaled Quality (QUAL)
Variants per Site
Nucleotide Content
Nucleotides
Annotations
Base pairs
0.0e+00
5.0e+06
1.0e+07
1.5e+07
2.0e+07
2.5e+07

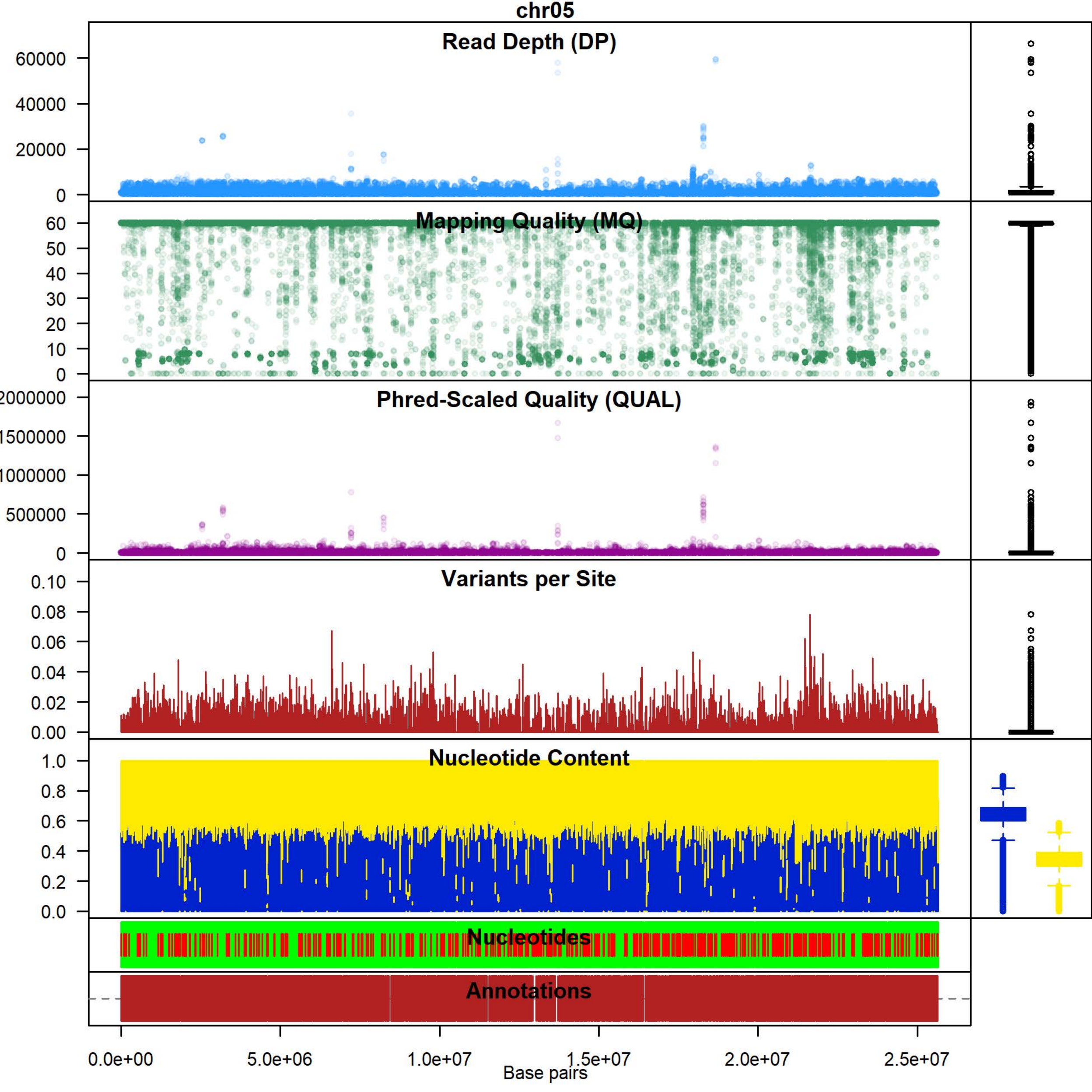
chr05
Read Depth (DP)
60000
40000
20000
0
Mapping Quality (MQ)
60
50
40
30
20
10
0
Phred-Scaled Quality (QUAL)
2000000
1500000
1000000
500000
0
Variants per Site
0.10
0.08
0.06
0.04
0.02
0.00
Nucleotide Content
1.0
0.8
0.6
0.4
0.2
0.0
Nucleotides
Annotations
0.0e+00
5.0e+06
1.0e+07
1.5e+07
2.0e+07
2.5e+07
Base pairs

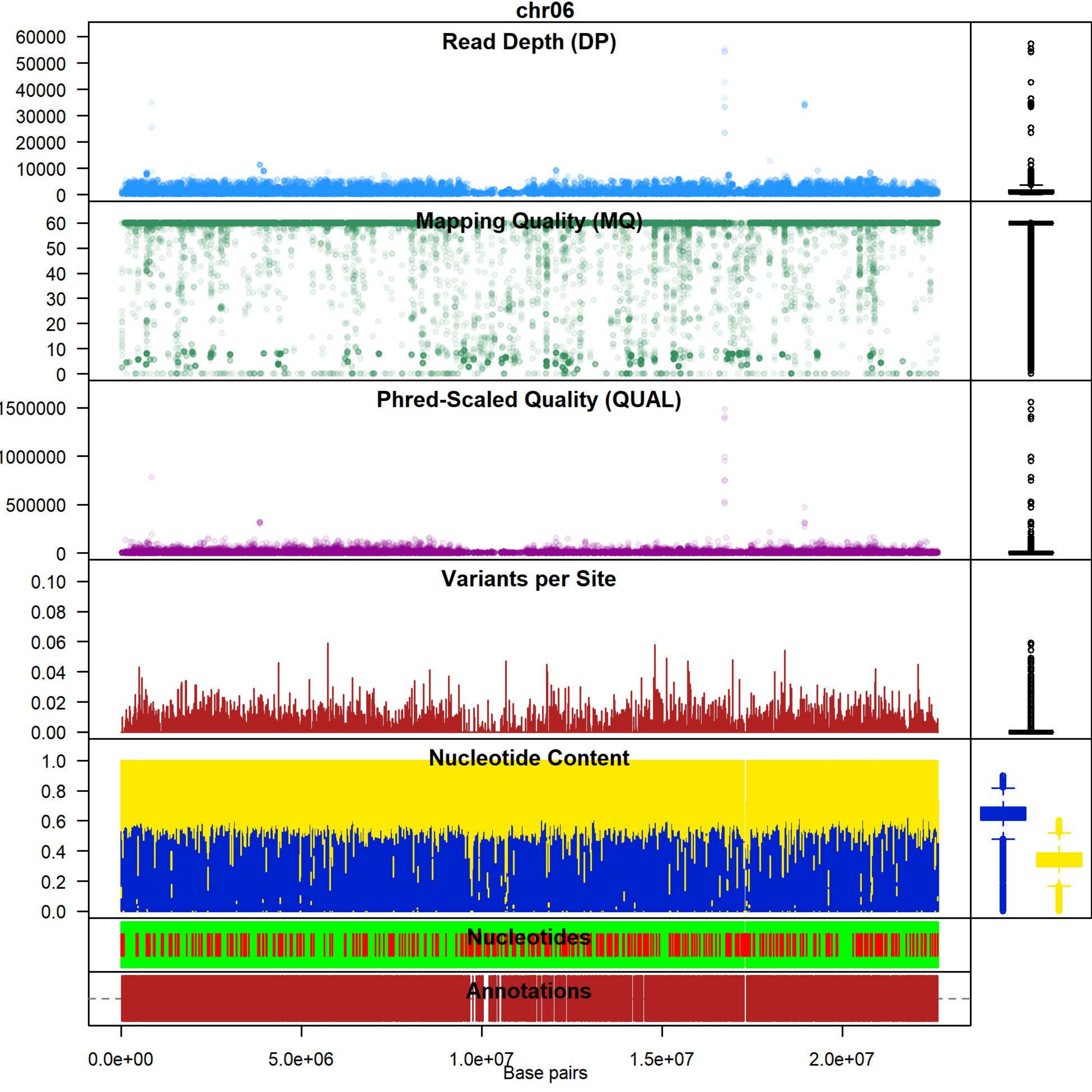

chr06
Read Depth (DP)
Mapping Quality (MQ)
Phred-Scaled Quality (QUAL)
Variants per Site
Nucleotide Content
Nucleotides
Annotations
Base pairs
0.0e+00
5.0e+06
1.0e+07
1.5e+07
2.0e+07

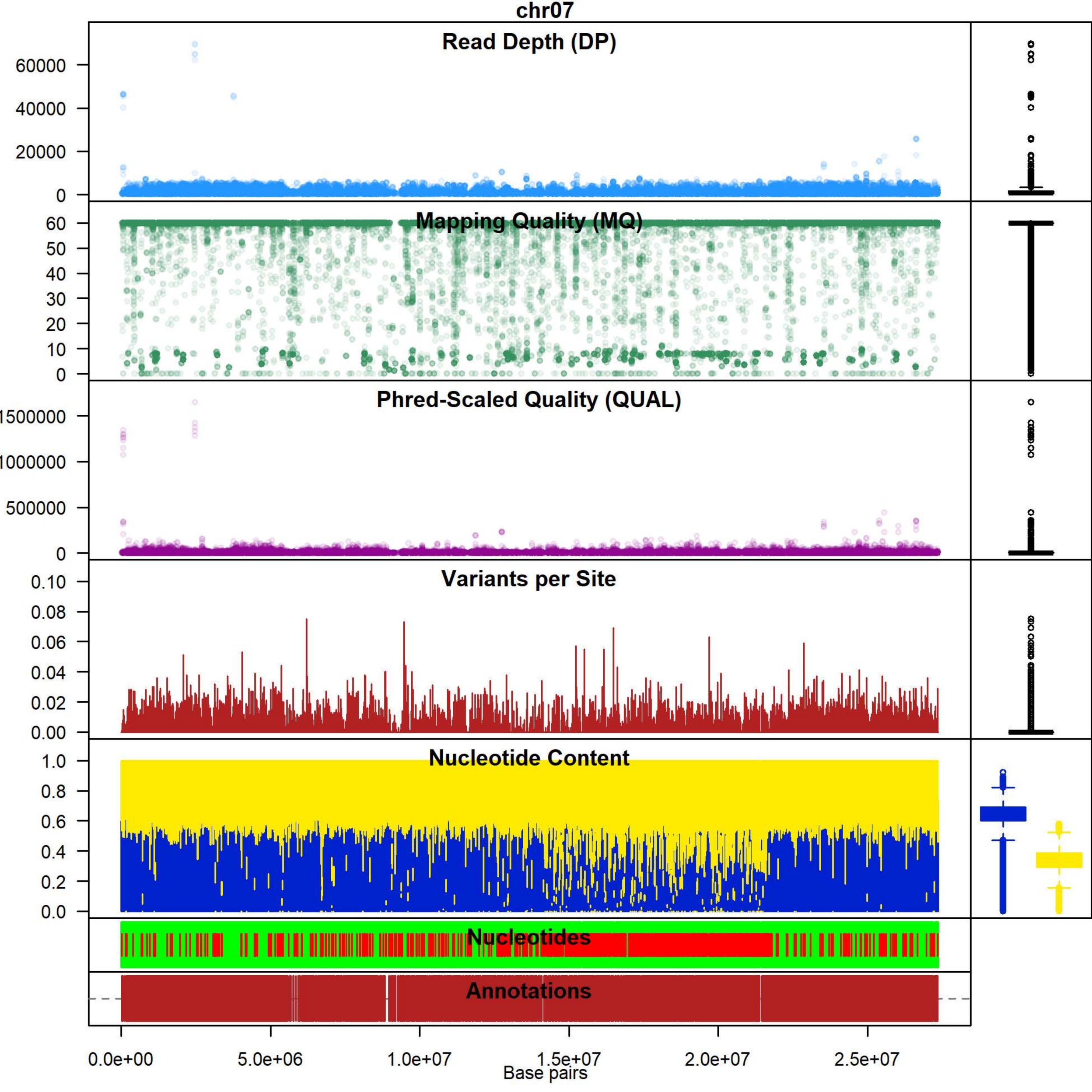

chr07
Read Depth (DP)
60000
40000
20000
0
Mapping Quality (MQ)
60
50
40
30
20
10
0
Phred-Scaled Quality (QUAL)
1500000
1000000
500000
0
Variants per Site
0.10
0.08
0.06
0.04
0.02
0.00
Nucleotide Content
1.0
0.8
0.6
0.4
0.2
0.0
Nucleotides
Annotations
0.0e+00
5.0e+06
1.0e+07
1.5e+07
2.0e+07
2.5e+07
Base pairs

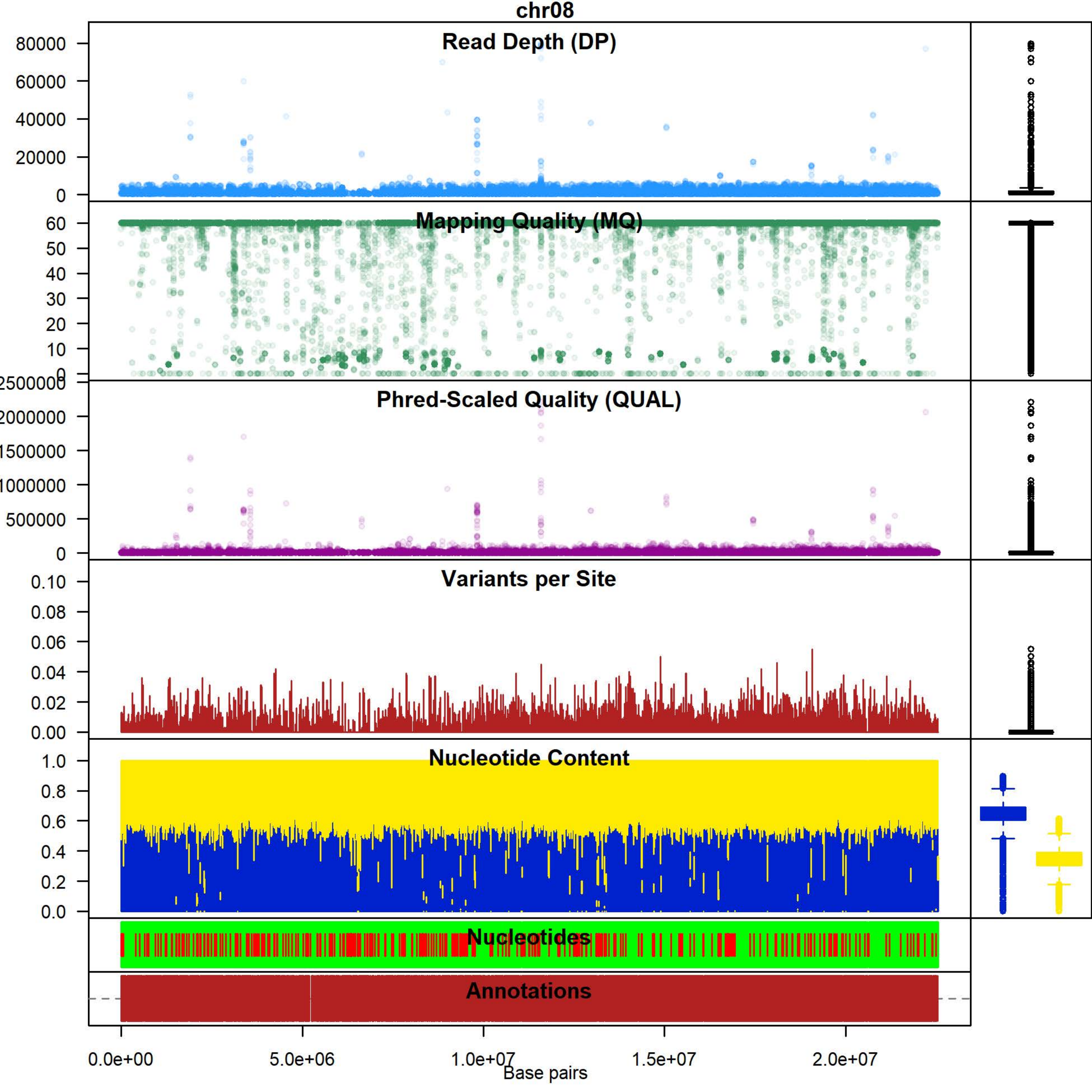

chr08
Read Depth (DP)
80000
60000
40000
20000
0
Mapping Quality (MQ)
60
50
40
30
20
10
0
Phred-Scaled Quality (QUAL)
2500000
2000000
1500000
1000000
500000
0
Variants per Site
0.10
0.08
0.06
0.04
0.02
0.00
Nucleotide Content
1.0
0.8
0.6
0.4
0.2
0.0
Nucleotides
Annotations
0.0e+00
5.0e+06
1.0e+07
1.5e+07
2.0e+07
Base pairs

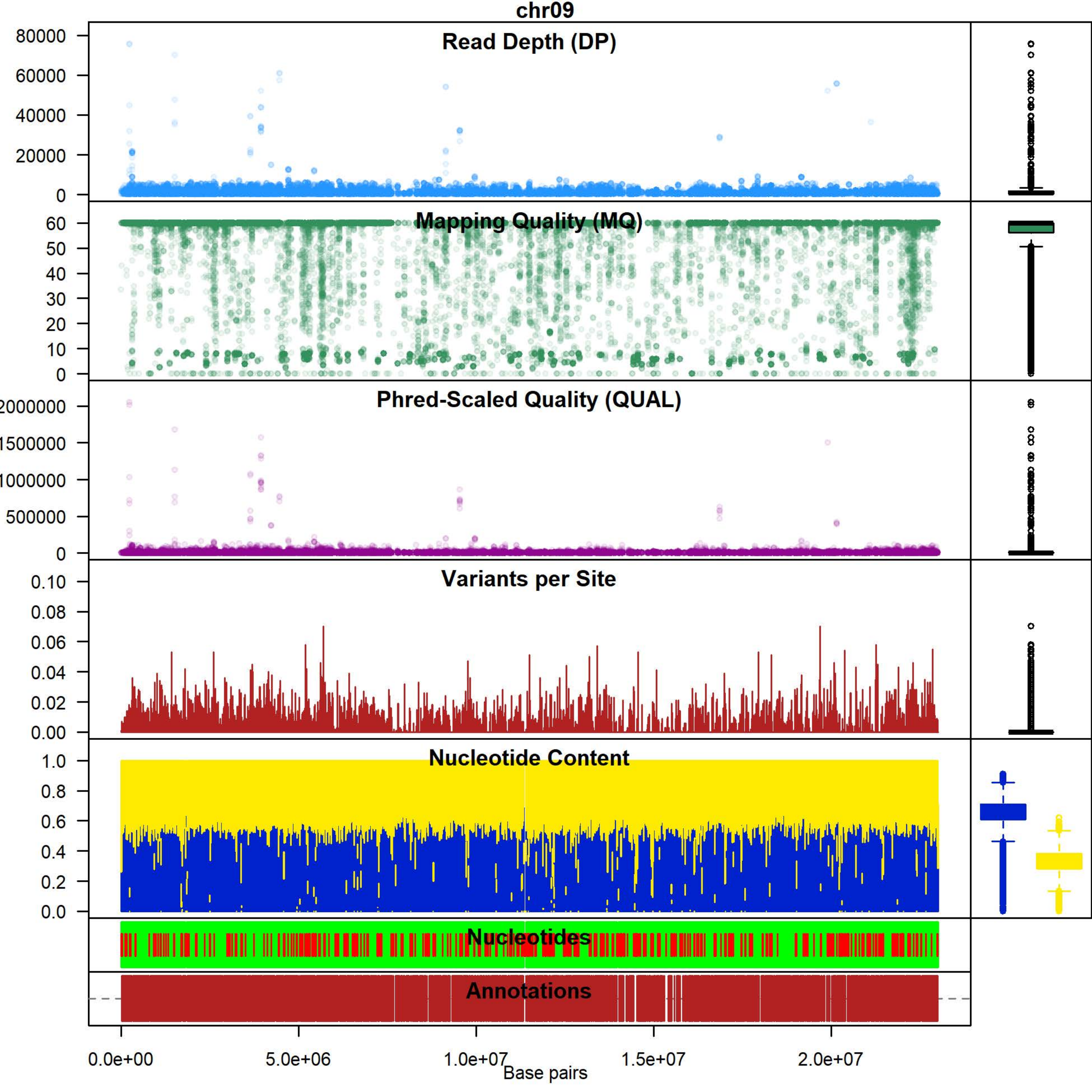
chr09
Read Depth (DP)
80000
60000
40000
20000
0
Mapping Quality (MQ)
60
50
40
30
20
10
0
Phred-Scaled Quality (QUAL)
2000000
1500000
1000000
500000
0
Variants per Site
0.10
0.08
0.06
0.04
0.02
0.00
Nucleotide Content
1.0
0.8
0.6
0.4
0.2
0.0
Nucleotides
Annotations
0.0e+00
5.0e+06
1.0e+07
1.5e+07
2.0e+07
Base pairs

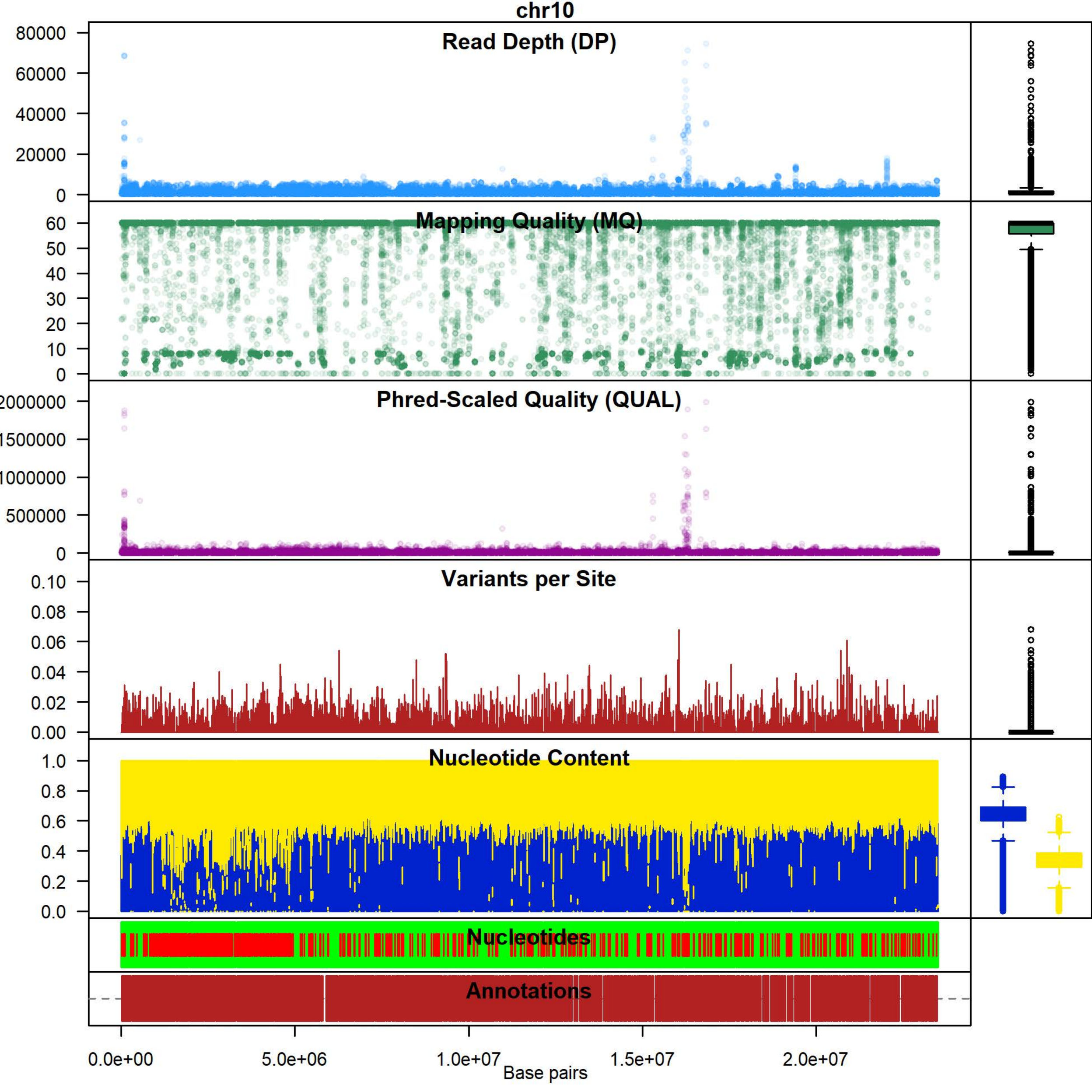
chr10
Read Depth (DP)
80000
60000
40000
20000
0
Mapping Quality (MQ)
60
50
40
30
20
10
0
Phred-Scaled Quality (QUAL)
2000000
1500000
1000000
500000
0
Variants per Site
0.10
0.08
0.06
0.04
0.02
0.00
Nucleotide Content
1.0
0.8
0.6
0.4
0.2
0.0
Nucleotides
Annotations
0.0e+00
5.0e+06
1.0e+07
1.5e+07
2.0e+07
Base pairs

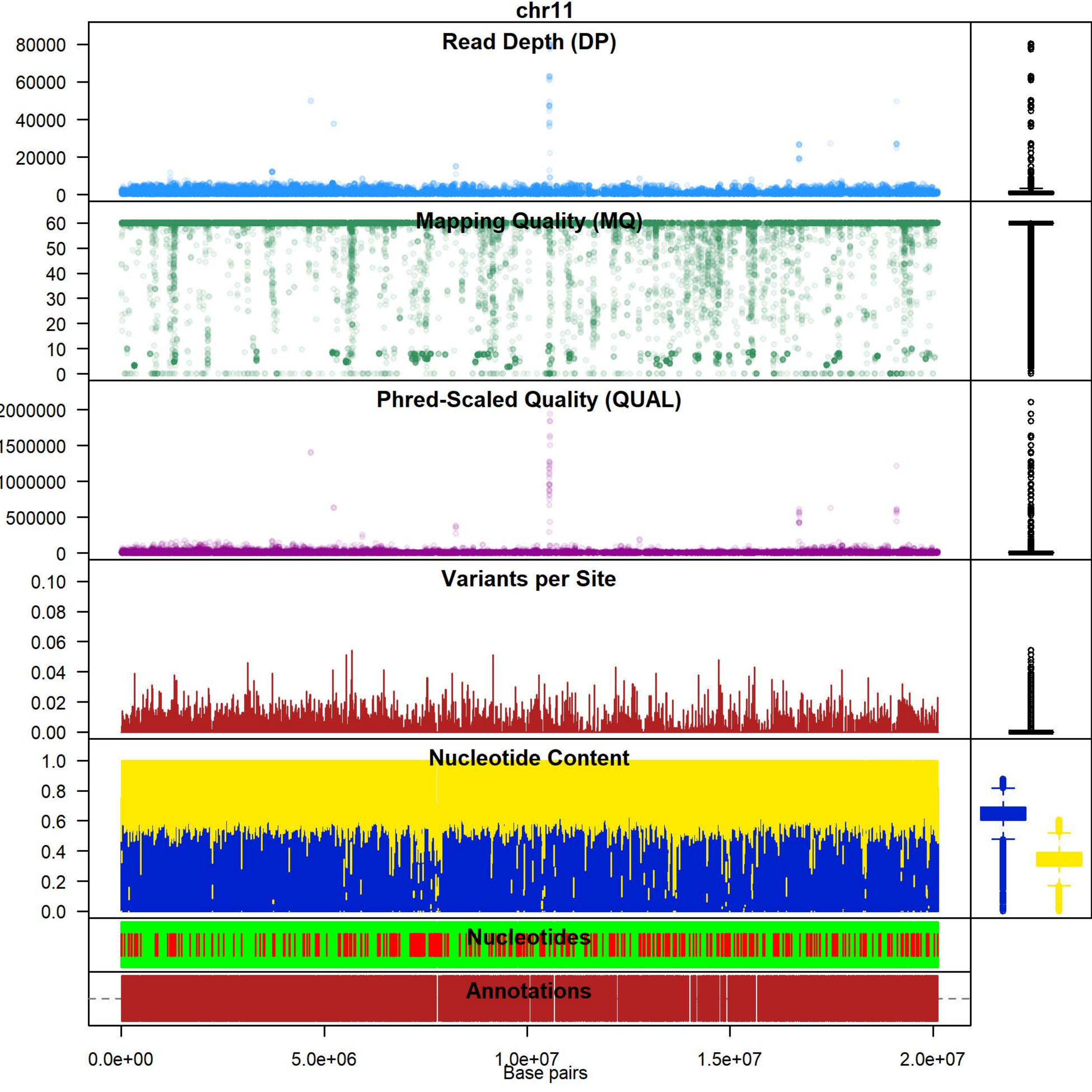

chr11
Read Depth (DP)
80000
60000
40000
20000
0
Mapping Quality (MQ)
60
50
40
30
20
10
0
Phred-Scaled Quality (QUAL)
2000000
1500000
1000000
500000
0
Variants per Site
0.10
0.08
0.06
0.04
0.02
0.00
Nucleotide Content
1.0
0.8
0.6
0.4
0.2
0.0
Nucleotides
Annotations
0.0e+00
5.0e+06
1.0e+07
1.5e+07
2.0e+07
Base pairs

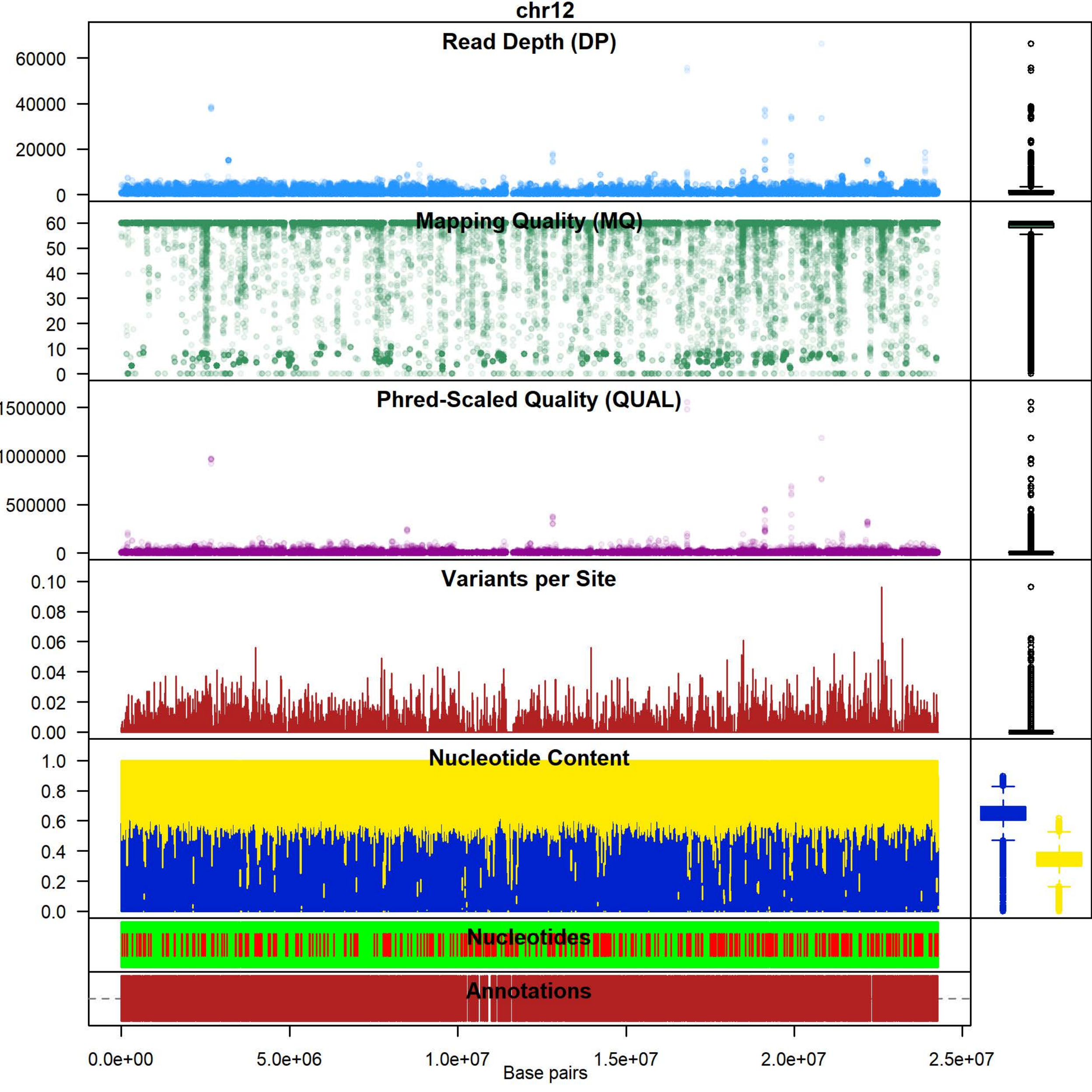

chr12
Read Depth (DP)
60000
40000
20000
0
Mapping Quality (MQ)
60
50
40
30
20
10
0
Phred-Scaled Quality (QUAL)
1500000
1000000
500000
0
Variants per Site
0.10
0.08
0.06
0.04
0.02
0.00
Nucleotide Content
1.0
0.8
0.6
0.4
0.2
0.0
Nucleotides
Annotations
0.0e+00
5.0e+06
1.0e+07
1.5e+07
2.0e+07
2.5e+07
Base pairs

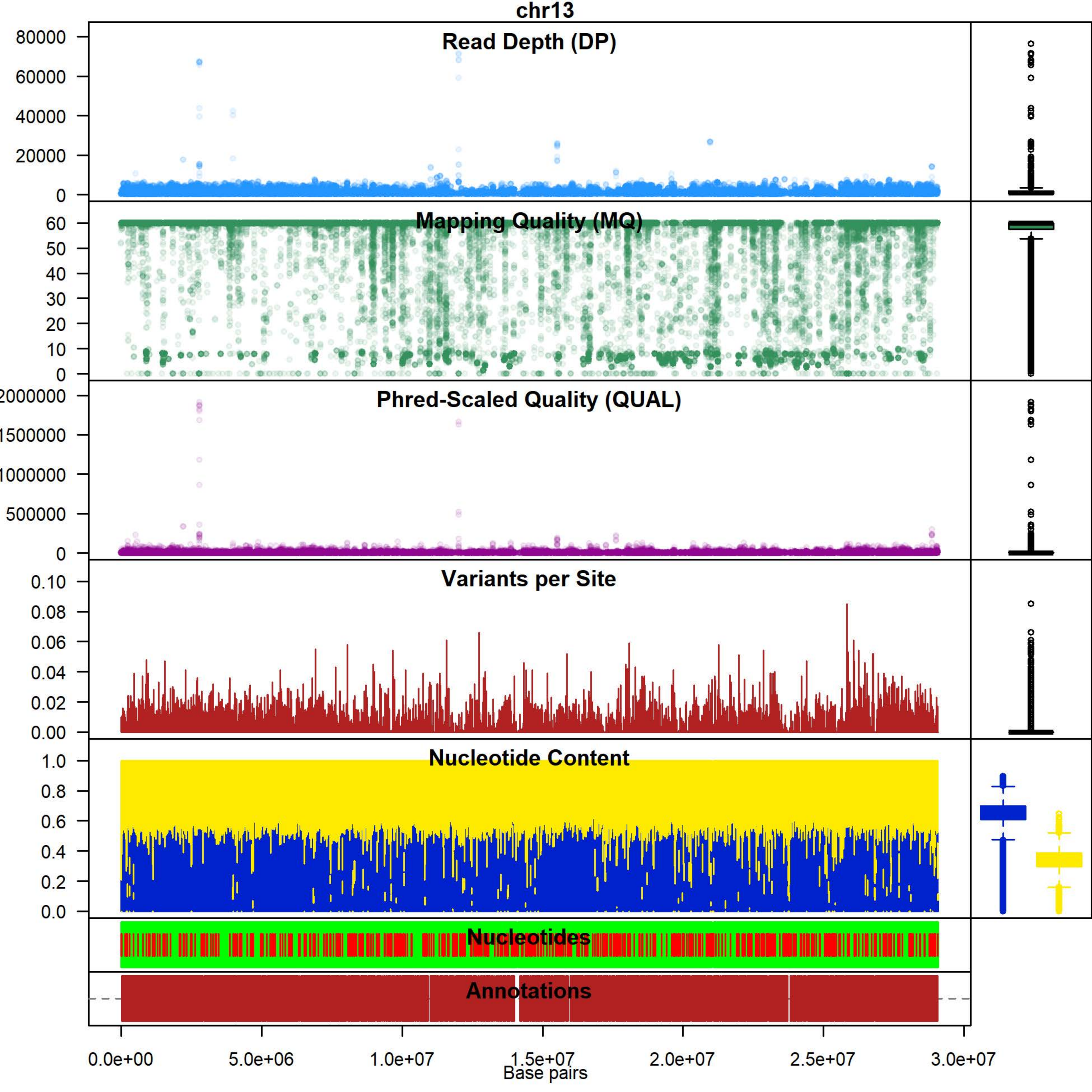
chr13
Read Depth (DP)
Mapping Quality (MQ)
Phred-Scaled Quality (QUAL)
Variants per Site
Nucleotide Content
Nucleotides
Annotations
Base pairs
80000
60000
40000
20000
0
60
50
40
30
20
10
0
2000000
1500000
1000000
500000
0
0.10
0.08
0.06
0.04
0.02
0.00
1.0
0.8
0.6
0.4
0.2
0.0
0.0e+00
5.0e+06
1.0e+07
1.5e+07
2.0e+07
2.5e+07
3.0e+07

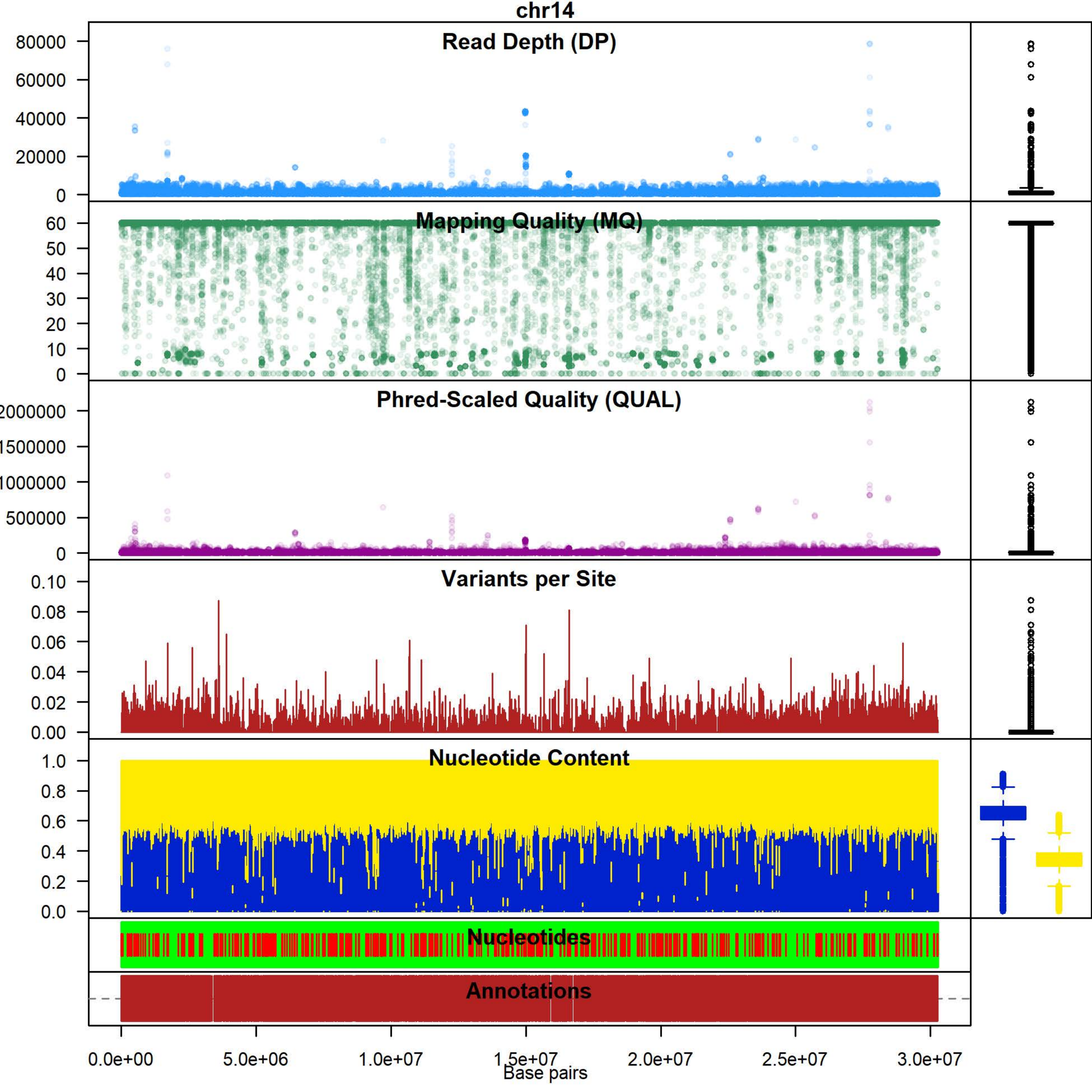

chr14
Read Depth (DP)
80000
60000
40000
20000
0
Mapping Quality (MQ)
60
50
40
30
20
10
0
Phred-Scaled Quality (QUAL)
2000000
1500000
1000000
500000
0
Variants per Site
0.10
0.08
0.06
0.04
0.02
0.00
Nucleotide Content
1.0
0.8
0.6
0.4
0.2
0.0
Nucleotides
Annotations
0.0e+00
5.0e+06
1.0e+07
1.5e+07
2.0e+07
2.5e+07
3.0e+07
Base pairs

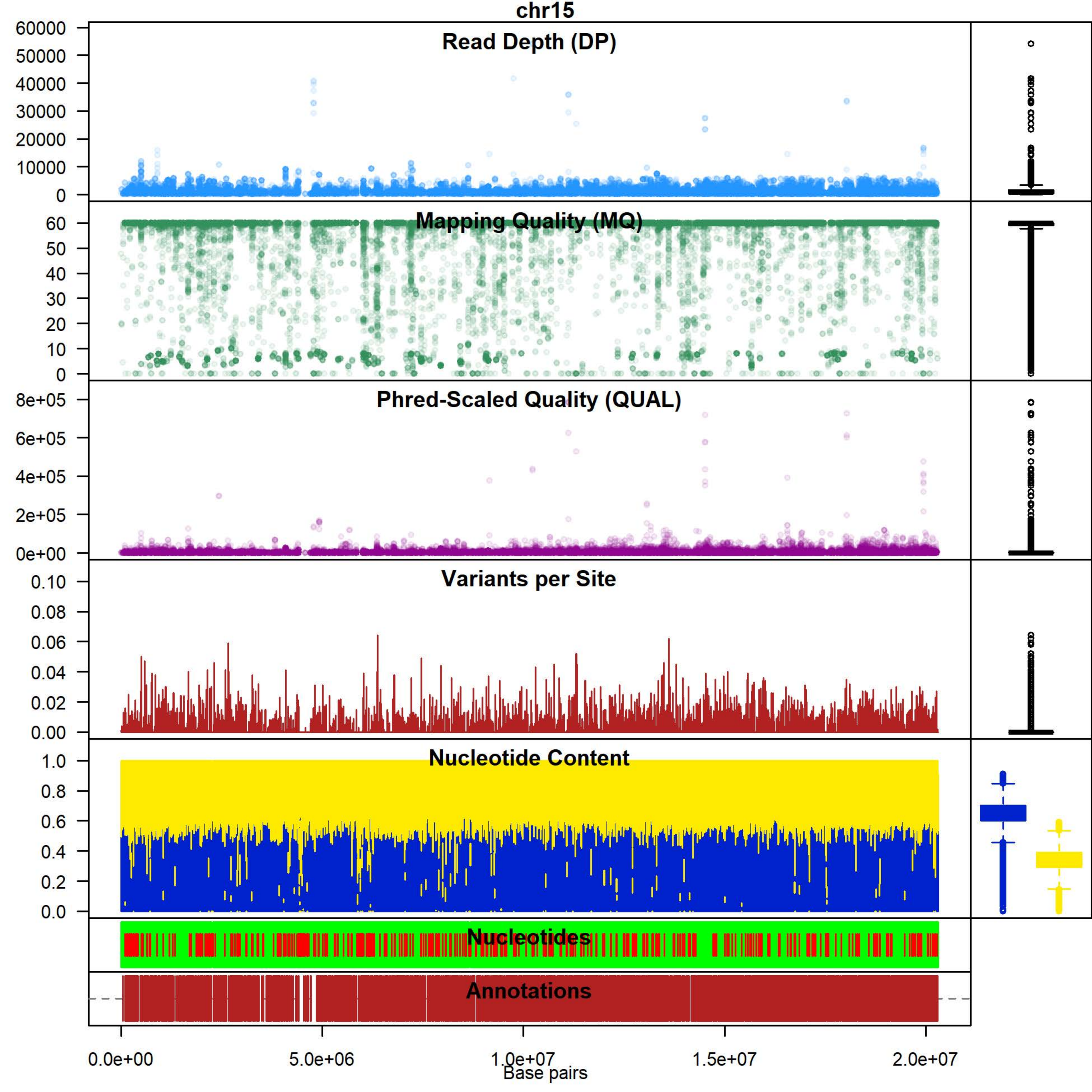

chr15
Read Depth (DP)
60000
50000
40000
30000
20000
10000
0
Mapping Quality (MQ)
60
50
40
30
20
10
0
Phred-Scaled Quality (QUAL)
8e+05
6e+05
4e+05
2e+05
0e+00
Variants per Site
0.10
0.08
0.06
0.04
0.02
0.00
Nucleotide Content
1.0
0.8
0.6
0.4
0.2
0.0
Nucleotides
Annotations
0.0e+00
5.0e+06
1.0e+07
1.5e+07
2.0e+07
Base pairs

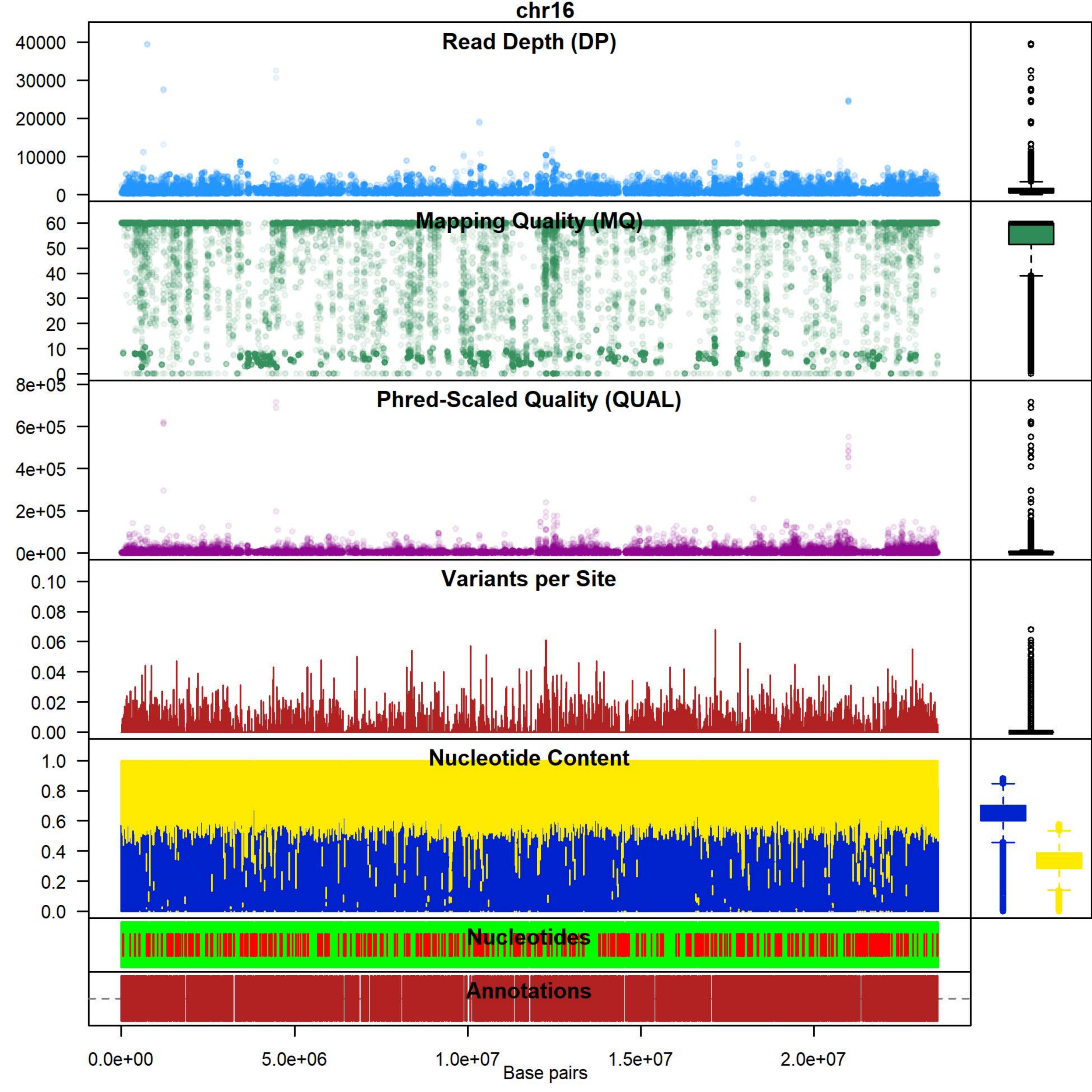
chr16
Read Depth (DP)
Mapping Quality (MQ)
Phred-Scaled Quality (QUAL)
Variants per Site
Nucleotide Content
Nucleotides
Annotations
Base pairs
0.0e+00
5.0e+06
1.0e+07
1.5e+07
2.0e+07
40000
30000
20000
10000
60
50
40
30
20
10
0
8e+05
6e+05
4e+05
2e+05
0e+00
0.10
0.08
0.06
0.04
0.02
0.00
1.0
0.8
0.6
0.4
0.2
0.0

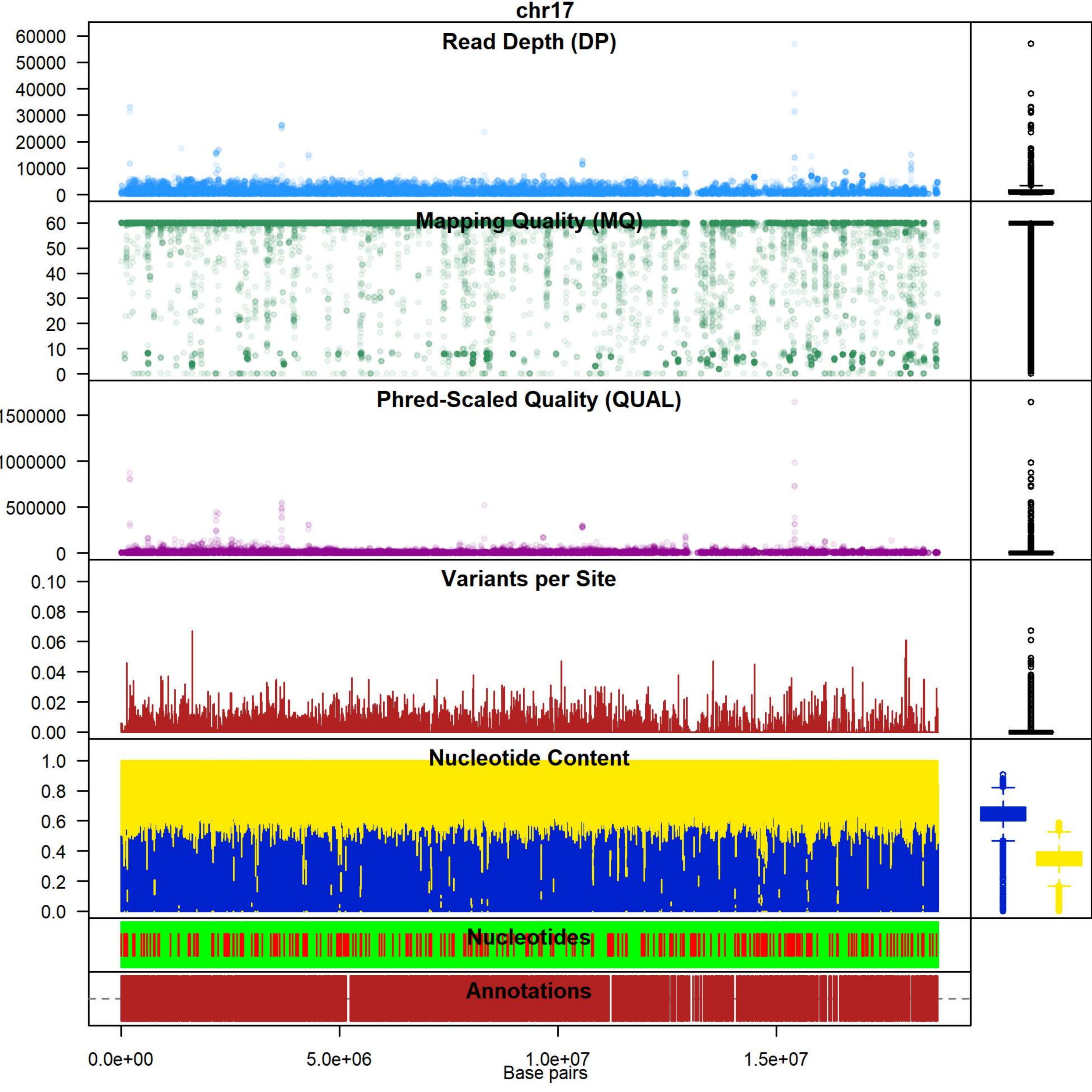
chr17
Read Depth (DP)
60000
50000
40000
30000
20000
10000
0
Mapping Quality (MQ)
60
50
40
30
20
10
0
Phred-Scaled Quality (QUAL)
1500000
1000000
500000
0
Variants per Site
0.10
0.08
0.06
0.04
0.02
0.00
Nucleotide Content
1.0
0.8
0.6
0.4
0.2
0.0
Nucleotides
Annotations
0.0e+00
5.0e+06
1.0e+07
1.5e+07
Base pairs

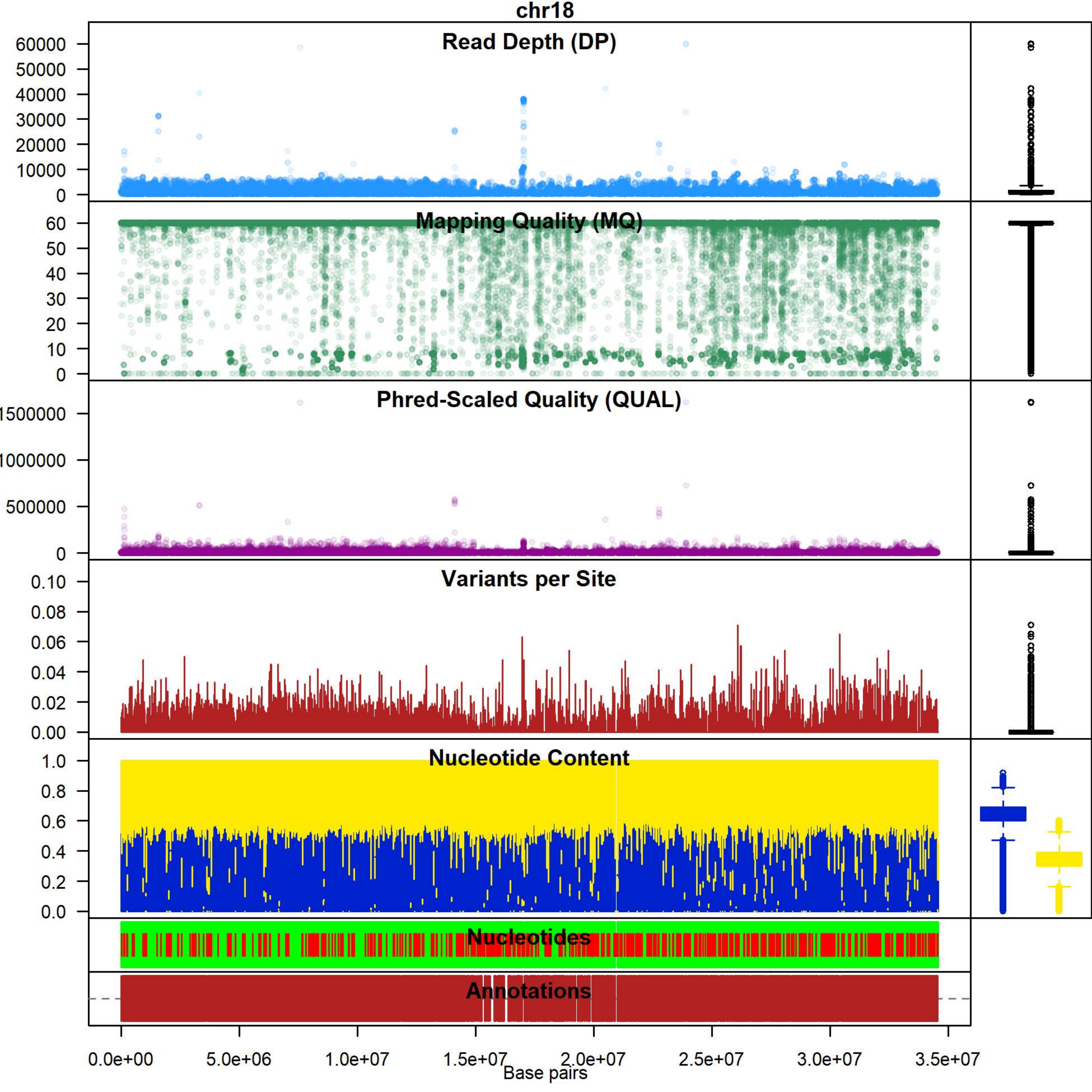
chr18
Read Depth (DP)
60000
50000
40000
30000
20000
10000
0
Mapping Quality (MQ)
60
50
40
30
20
10
0
Phred-Scaled Quality (QUAL)
1500000
1000000
500000
0
Variants per Site
0.10
0.08
0.06
0.04
0.02
0.00
Nucleotide Content
1.0
0.8
0.6
0.4
0.2
0.0
Nucleotides
Annotations
0.0e+00
5.0e+06
1.0e+07
1.5e+07
2.0e+07
2.5e+07
3.0e+07
3.5e+07
Base pairs

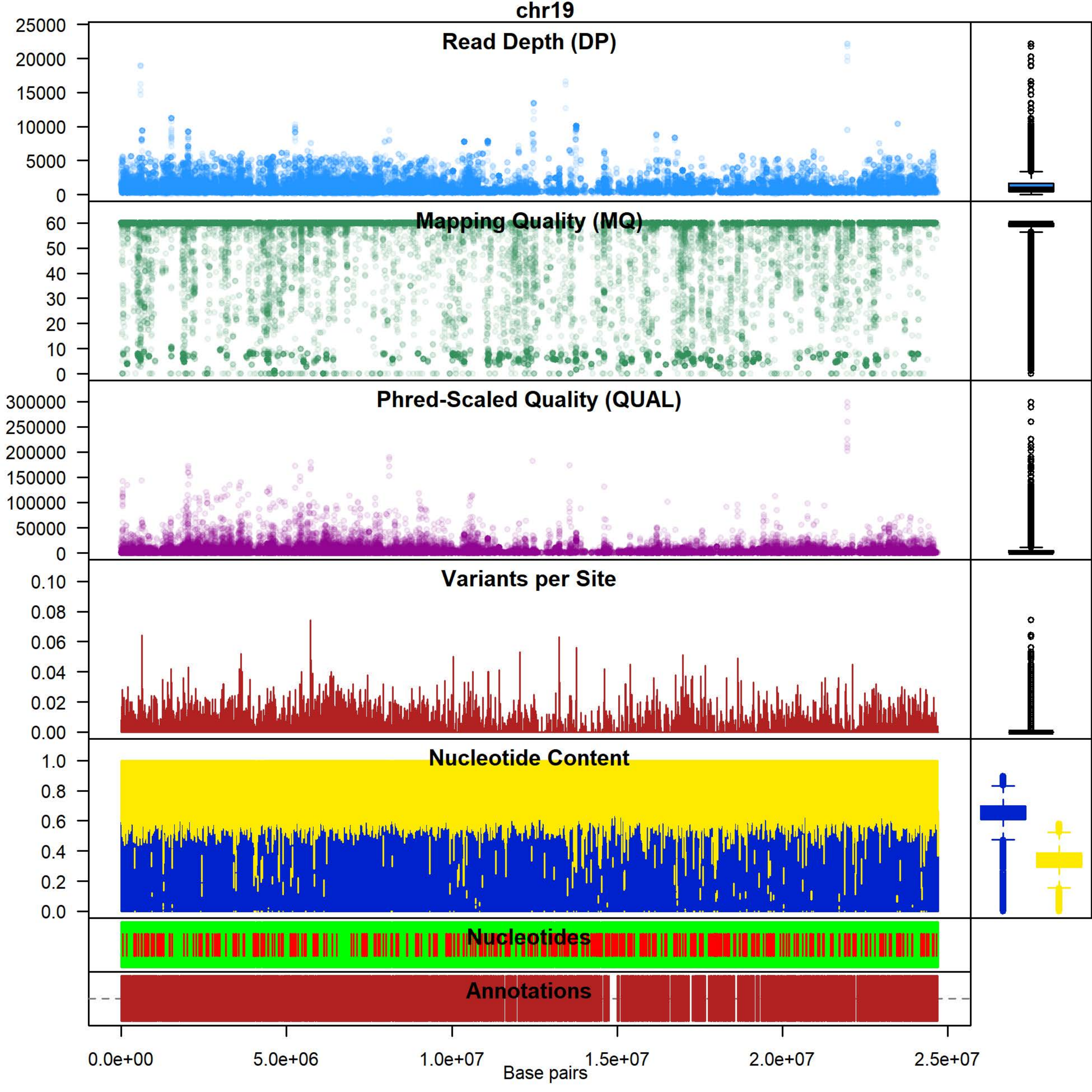

chr19
Read Depth (DP)
Mapping Quality (MQ)
Phred-Scaled Quality (QUAL)
Variants per Site
Nucleotide Content
Nucleotides
Annotations
Base pairs
0.0e+00
5.0e+06
1.0e+07
1.5e+07
2.0e+07
2.5e+07
