# Supplementary material for: Characterization of Bunch Compactness in a Diverse Collection of Vitis vinifera L. Genotypes Enriched in Table Grape Cultivars Reveals New Candidate Genes Associated with Berry Number
Source: Plants (Basel). 2025 Apr 26;14(9):1308. doi: 10.3390/plants14091308 (PMC12073236; doi:10.3390/plants14091308)
Supplement: Supplementary file 1 [file plants-14-01308-s001.zip › Supplementary File S2 - QC inspection after filtering.pdf]

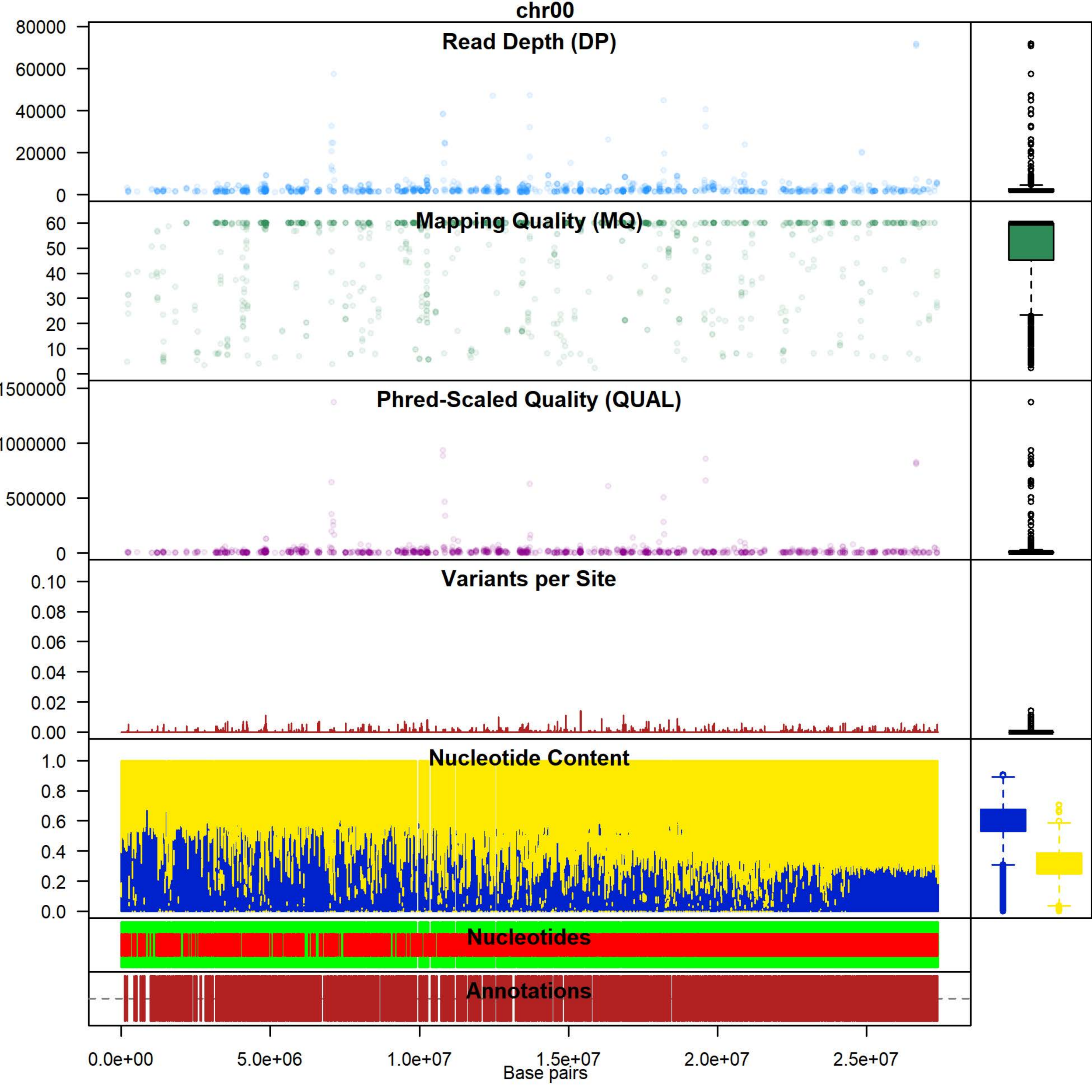
chr00
Read Depth (DP)
80000
60000
40000
20000
0
Mapping Quality (MQ)
60
50
40
30
20
10
0
Phred-Scaled Quality (QUAL)
1500000
1000000
500000
0
Variants per Site
0.10
0.08
0.06
0.04
0.02
0.00
Nucleotide Content
1.0
0.8
0.6
0.4
0.2
0.0
Nucleotides
Annotations
0.0e+00
5.0e+06
1.0e+07
1.5e+07
2.0e+07
2.5e+07
Base pairs

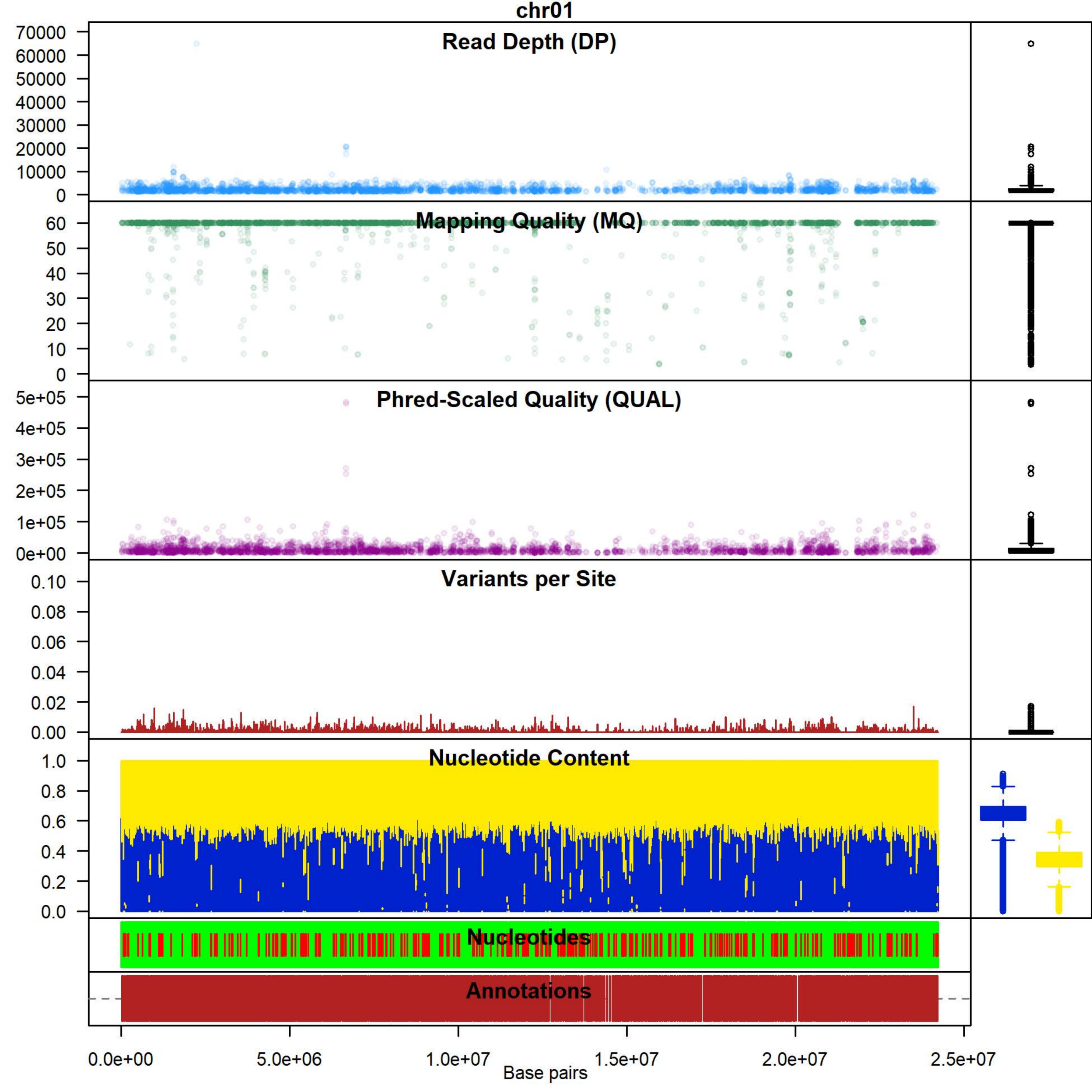
chr01
Read Depth (DP)
70000
60000
50000
40000
30000
20000
10000
0
Mapping Quality (MQ)
60
50
40
30
20
10
0
Phred-Scaled Quality (QUAL)
5e+05
4e+05
3e+05
2e+05
1e+05
0e+00
Variants per Site
0.10
0.08
0.06
0.04
0.02
0.00
Nucleotide Content
1.0
0.8
0.6
0.4
0.2
0.0
Nucleotides
Annotations
0.0e+00
5.0e+06
1.0e+07
1.5e+07
2.0e+07
2.5e+07
Base pairs

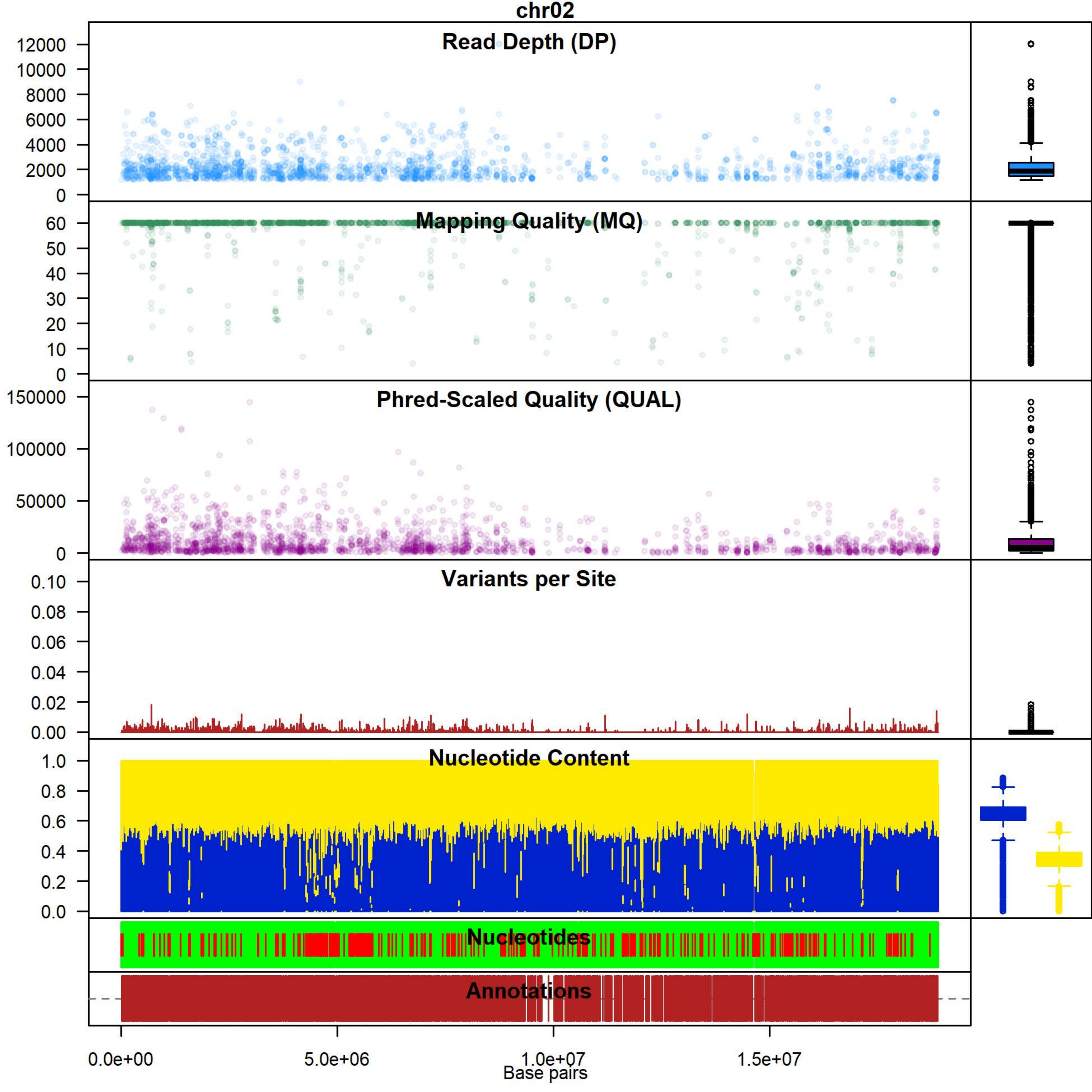

chr02
Read Depth (DP)
12000
10000
8000
6000
4000
2000
0
Mapping Quality (MQ)
60
50
40
30
20
10
0
Phred-Scaled Quality (QUAL)
150000
100000
50000
0
Variants per Site
0.10
0.08
0.06
0.04
0.02
0.00
Nucleotide Content
1.0
0.8
0.6
0.4
0.2
0.0
Nucleotides
Annotations
0.0e+00
5.0e+06
1.0e+07
1.5e+07
Base pairs

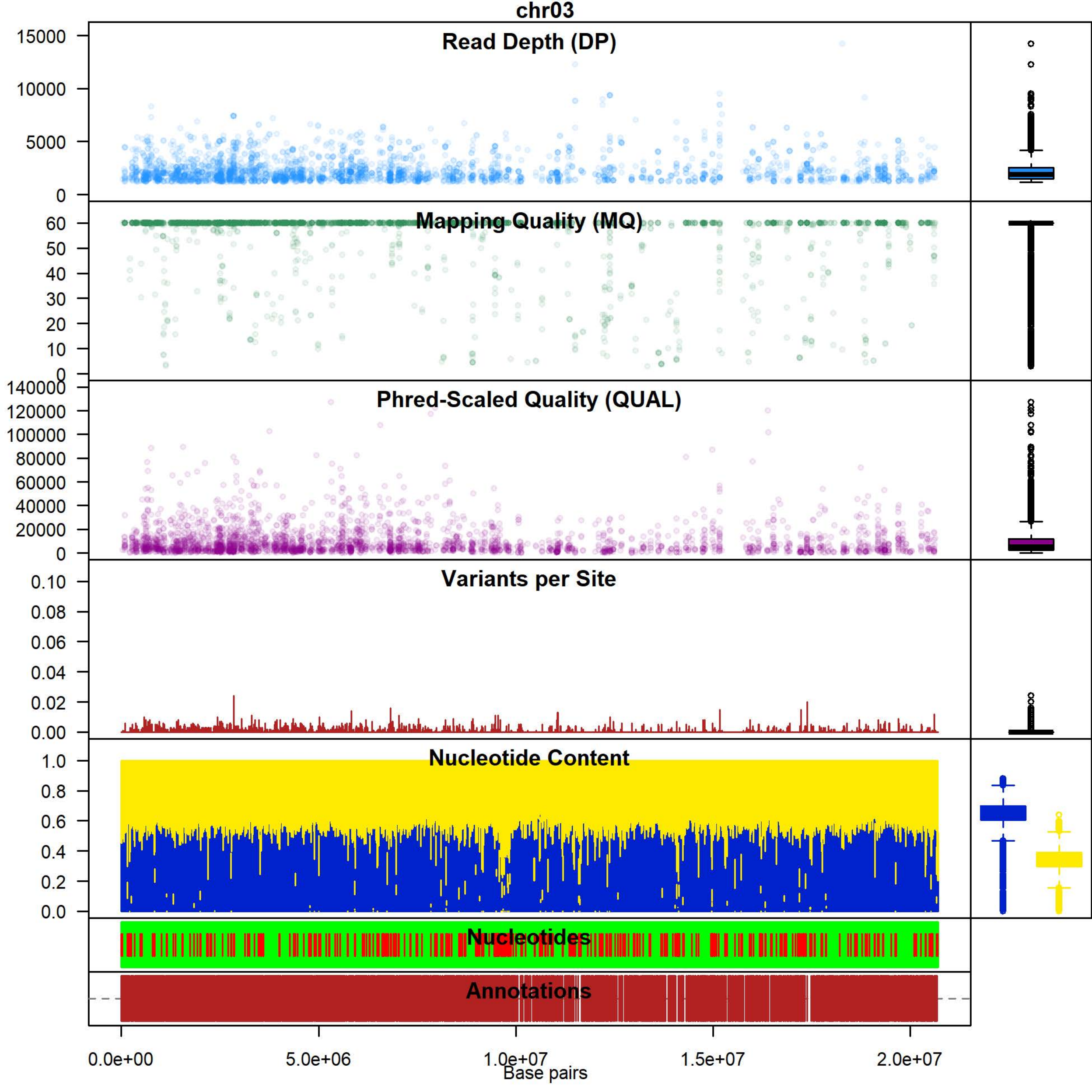
chr03
Read Depth (DP)
15000
10000
5000
0
Mapping Quality (MQ)
60
50
40
30
20
10
0
Phred-Scaled Quality (QUAL)
140000
120000
100000
80000
60000
40000
20000
0
Variants per Site
0.10
0.08
0.06
0.04
0.02
0.00
Nucleotide Content
1.0
0.8
0.6
0.4
0.2
0.0
Nucleotides
Annotations
0.0e+00
5.0e+06
1.0e+07
1.5e+07
2.0e+07
Base pairs

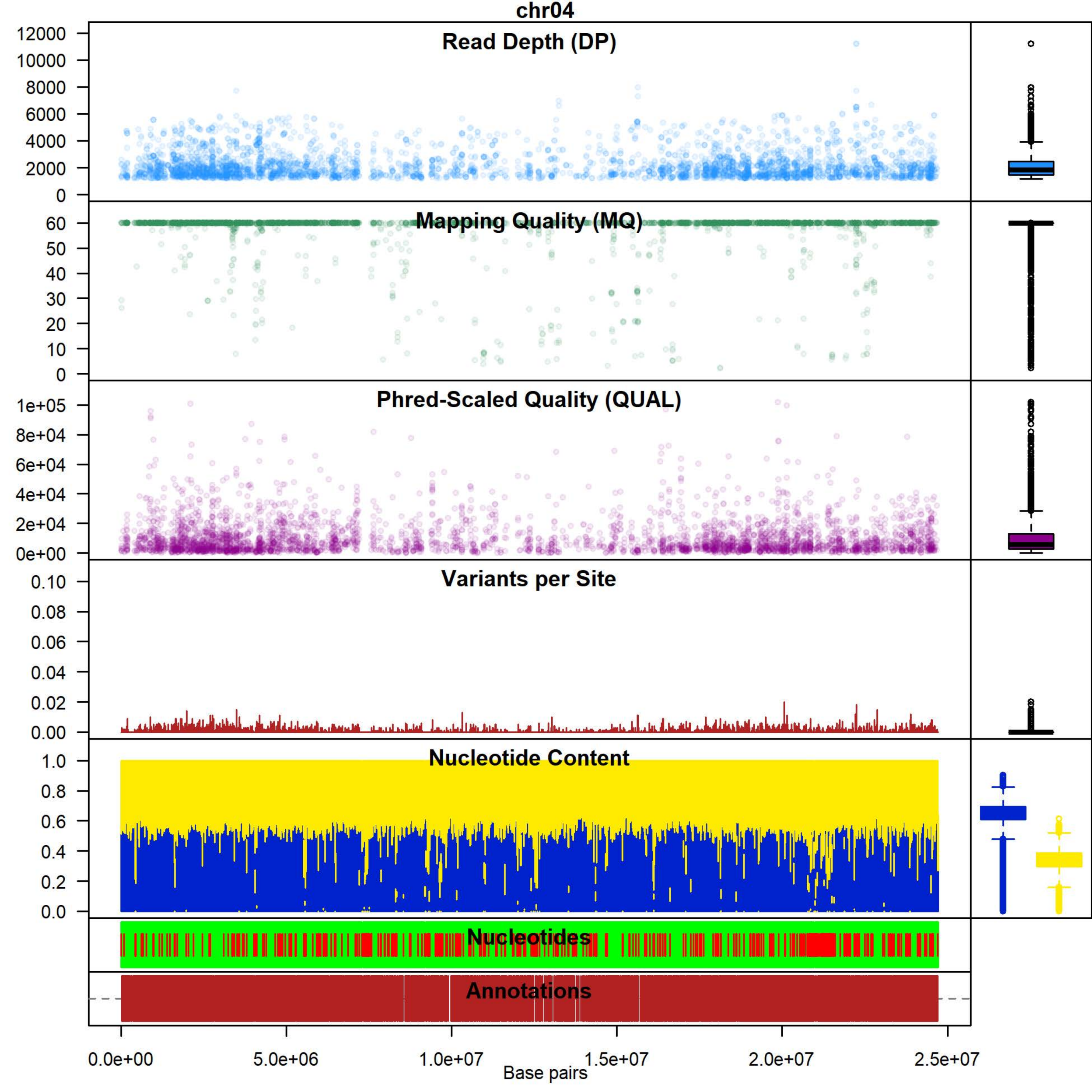

chr04
Read Depth (DP)
Mapping Quality (MQ)
Phred-Scaled Quality (QUAL)
Variants per Site
Nucleotide Content
Nucleotides
Annotations
Base pairs

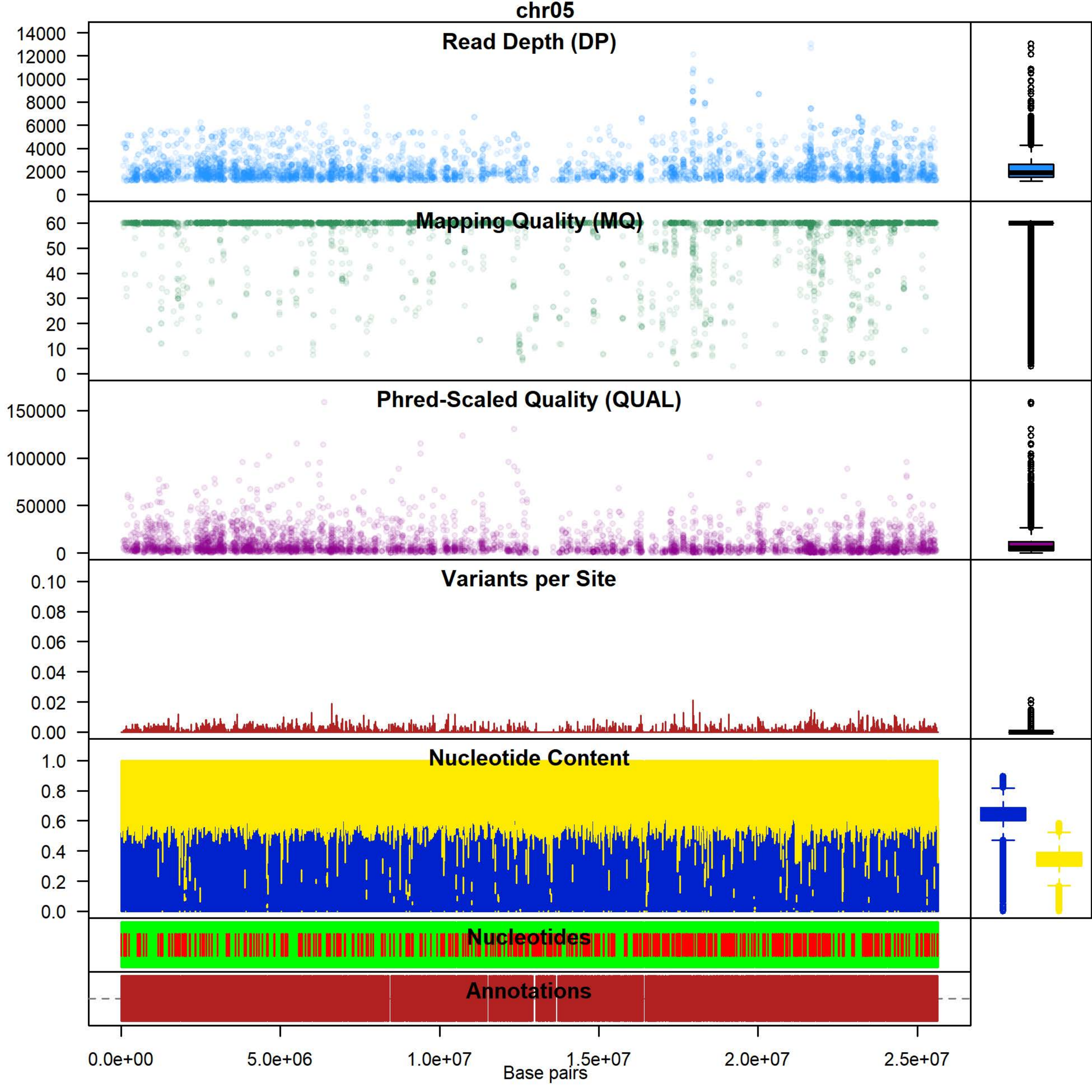

chr05
Read Depth (DP)
Mapping Quality (MQ)
Phred-Scaled Quality (QUAL)
Variants per Site
Nucleotide Content
Nucleotides
Annotations
Base pairs
0.0e+00
5.0e+06
1.0e+07
1.5e+07
2.0e+07
2.5e+07

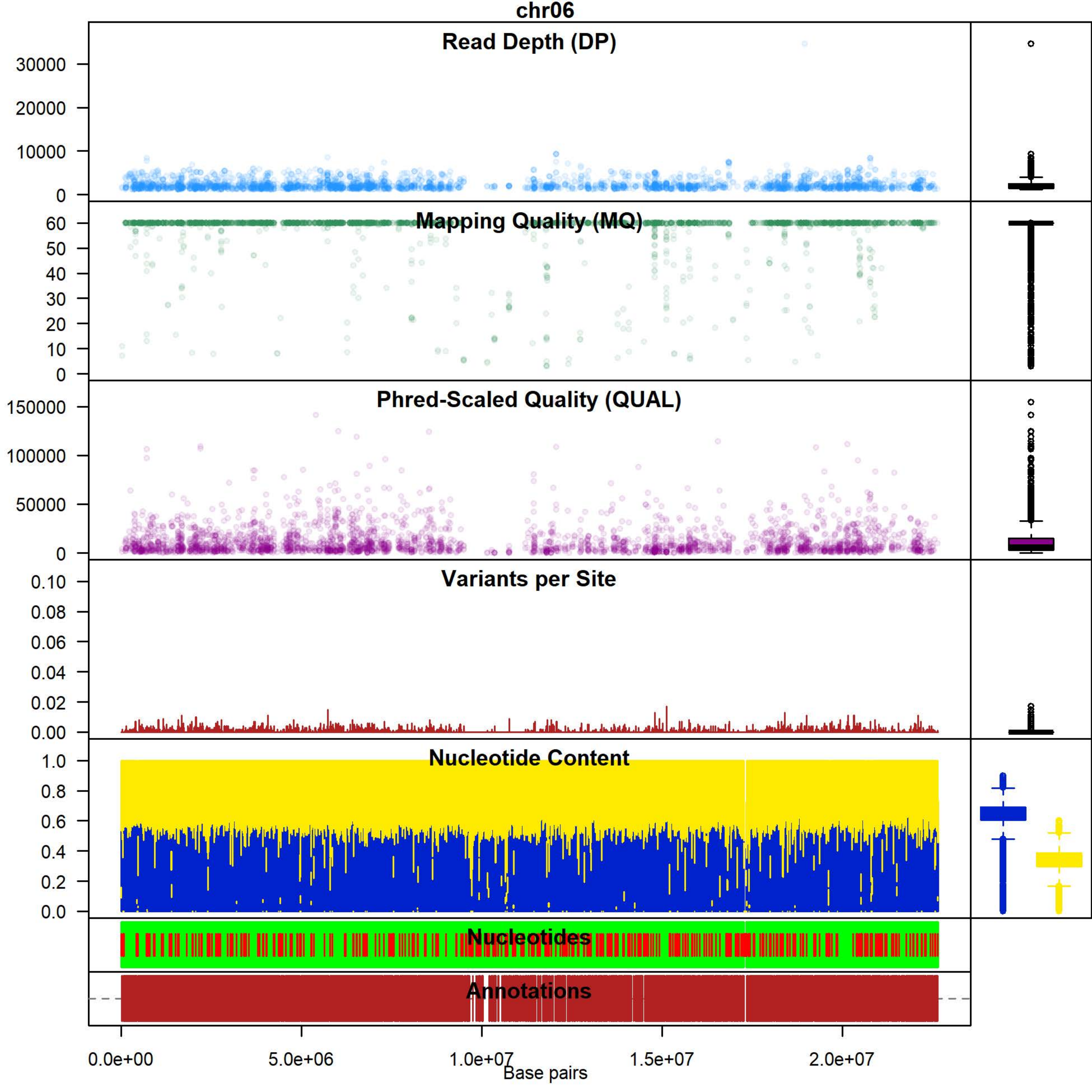

chr06
Read Depth (DP)
30000
20000
10000
0
Mapping Quality (MQ)
60
50
40
30
20
10
0
Phred-Scaled Quality (QUAL)
150000
100000
50000
0
Variants per Site
0.10
0.08
0.06
0.04
0.02
0.00
Nucleotide Content
1.0
0.8
0.6
0.4
0.2
0.0
Nucleotides
Annotations
0.0e+00
5.0e+06
1.0e+07
1.5e+07
2.0e+07
Base pairs

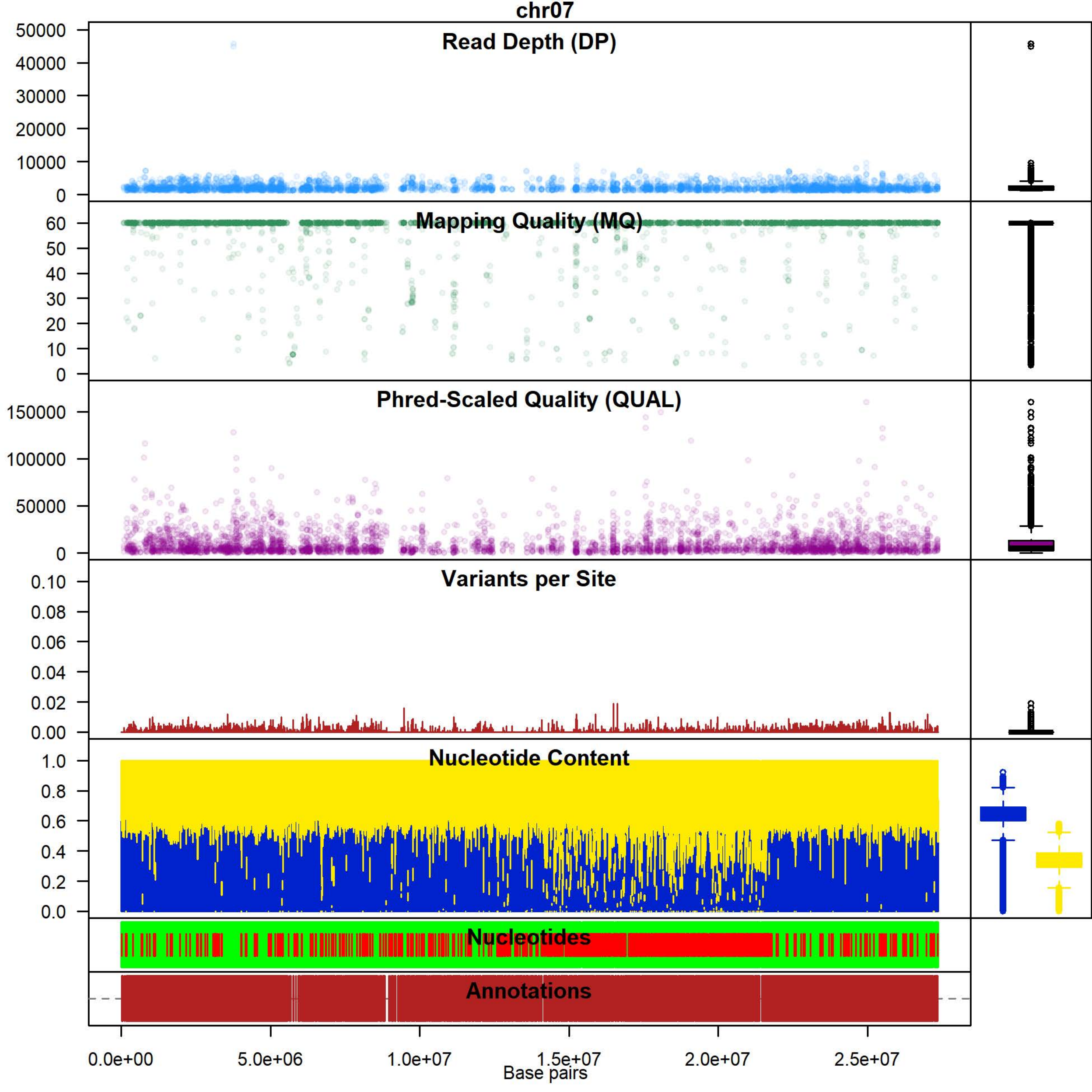
chr07
Read Depth (DP)
Mapping Quality (MQ)
Phred-Scaled Quality (QUAL)
Variants per Site
Nucleotide Content
Nucleotides
Annotations
Base pairs
0.0e+00
5.0e+06
1.0e+07
1.5e+07
2.0e+07
2.5e+07
50000
40000
30000
20000
10000
0
60
50
40
30
20
10
0
150000
100000
50000
0
0.10
0.08
0.06
0.04
0.02
0.00
1.0
0.8
0.6
0.4
0.2
0.0

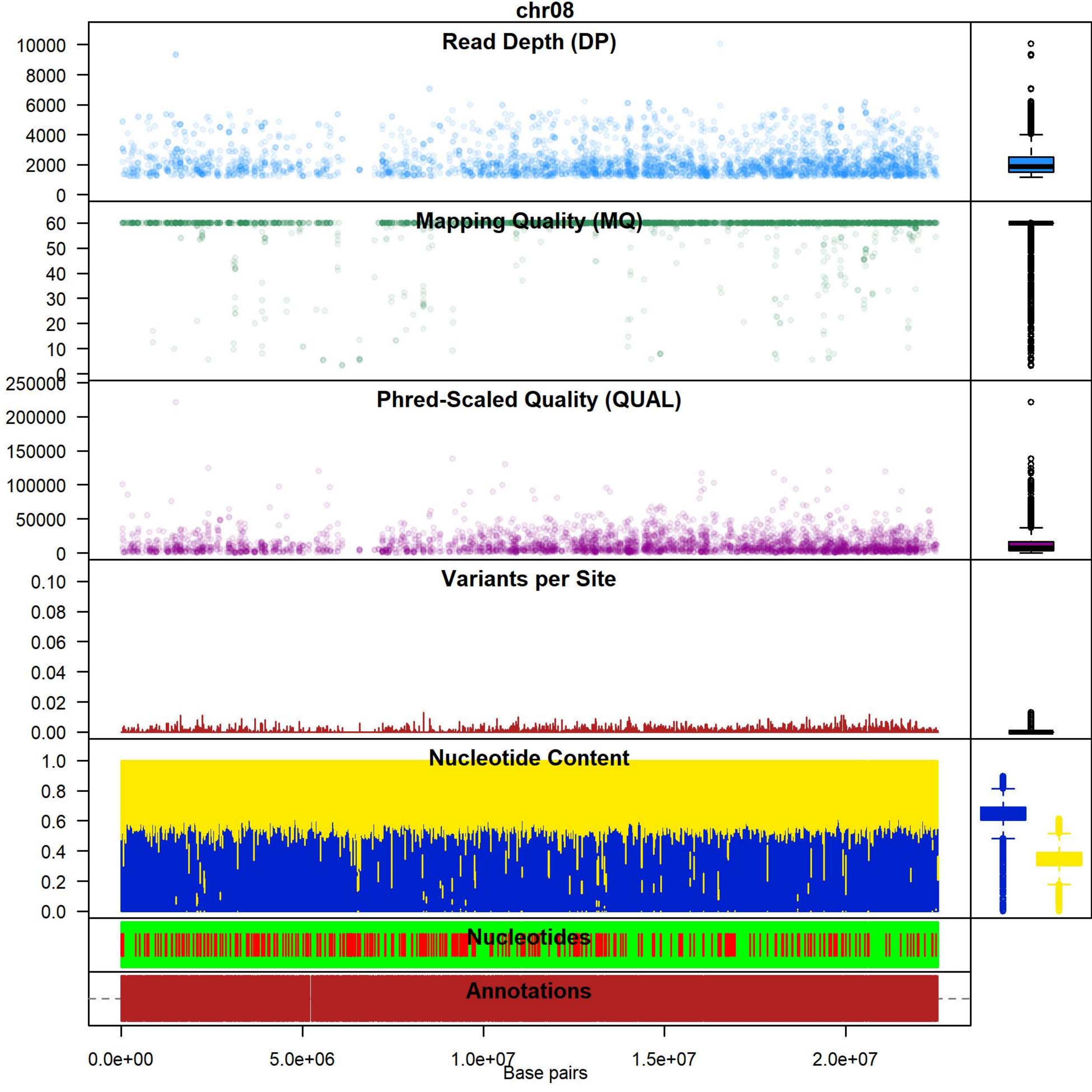

chr08
Read Depth (DP)
10000
8000
6000
4000
2000
0
Mapping Quality (MQ)
60
50
40
30
20
10
0
Phred-Scaled Quality (QUAL)
250000
200000
150000
100000
50000
0
Variants per Site
0.10
0.08
0.06
0.04
0.02
0.00
Nucleotide Content
1.0
0.8
0.6
0.4
0.2
0.0
Nucleotides
Annotations
0.0e+00
5.0e+06
1.0e+07
1.5e+07
2.0e+07
Base pairs

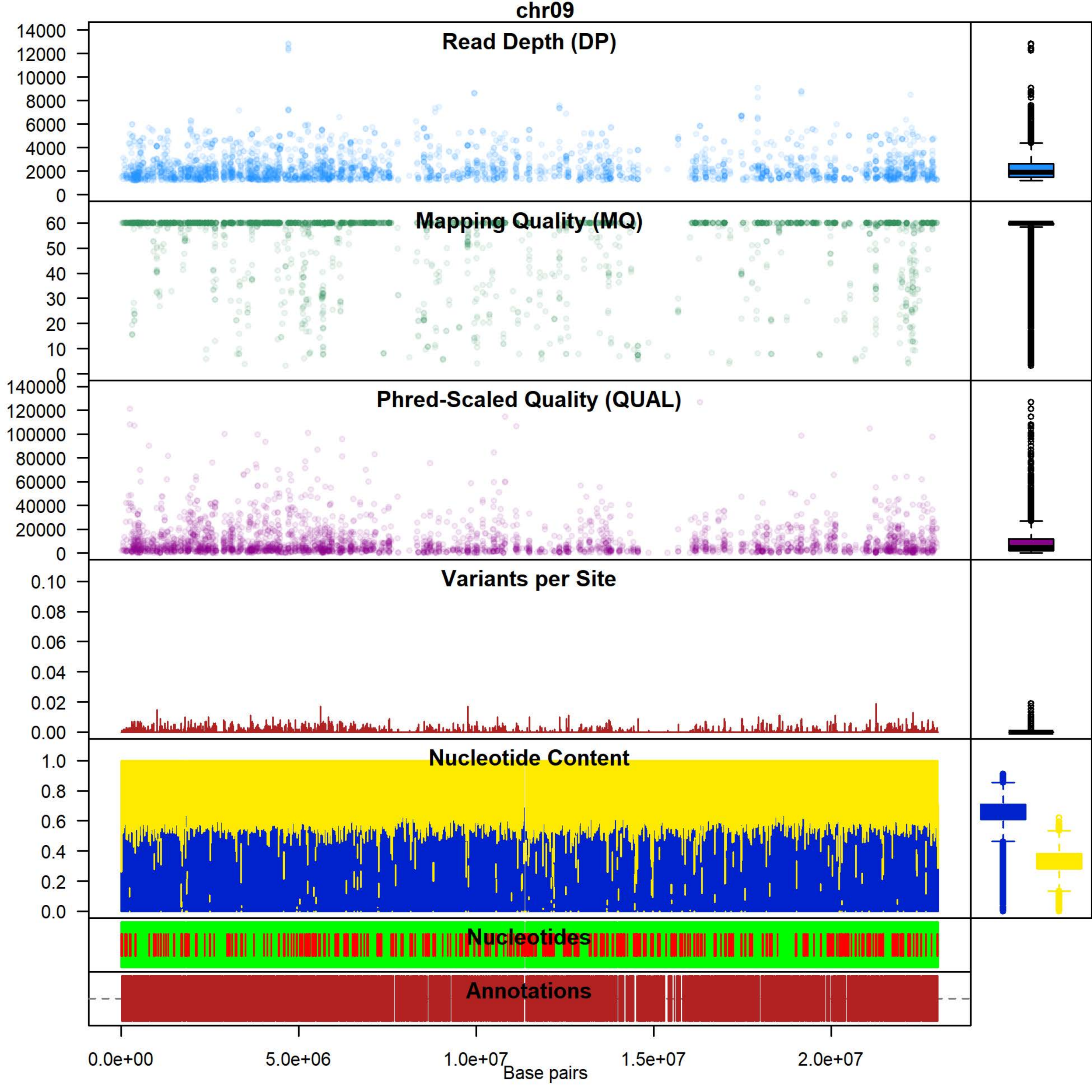
chr09
Read Depth (DP)
Mapping Quality (MQ)
Phred-Scaled Quality (QUAL)
Variants per Site
Nucleotide Content
Nucleotides
Annotations
Base pairs

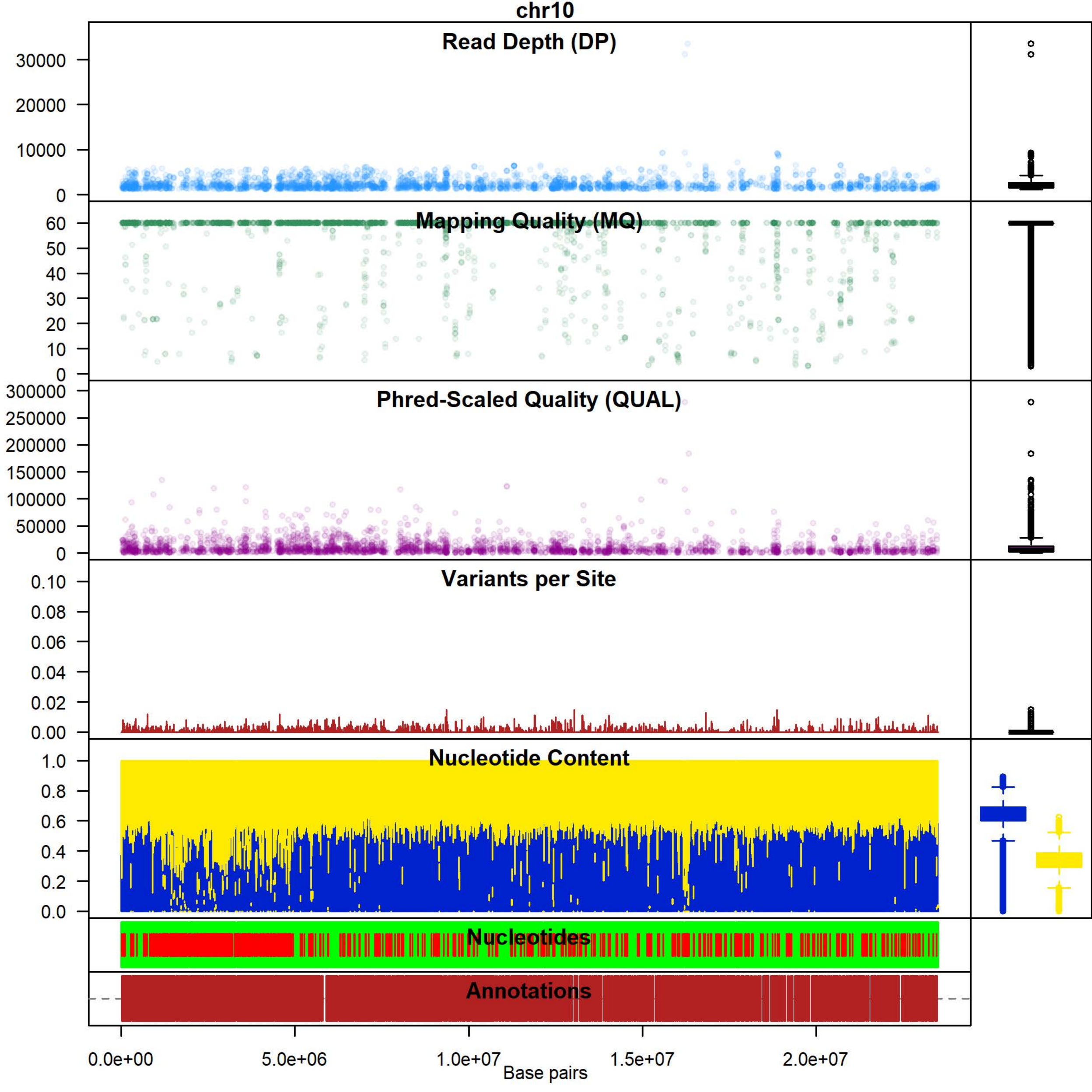
chr10
Read Depth (DP)
30000
20000
10000
0
Mapping Quality (MQ)
60
50
40
30
20
10
0
Phred-Scaled Quality (QUAL)
300000
250000
200000
150000
100000
50000
0
Variants per Site
0.10
0.08
0.06
0.04
0.02
0.00
Nucleotide Content
1.0
0.8
0.6
0.4
0.2
0.0
Nucleotides
Annotations
0.0e+00
5.0e+06
1.0e+07
1.5e+07
2.0e+07
Base pairs

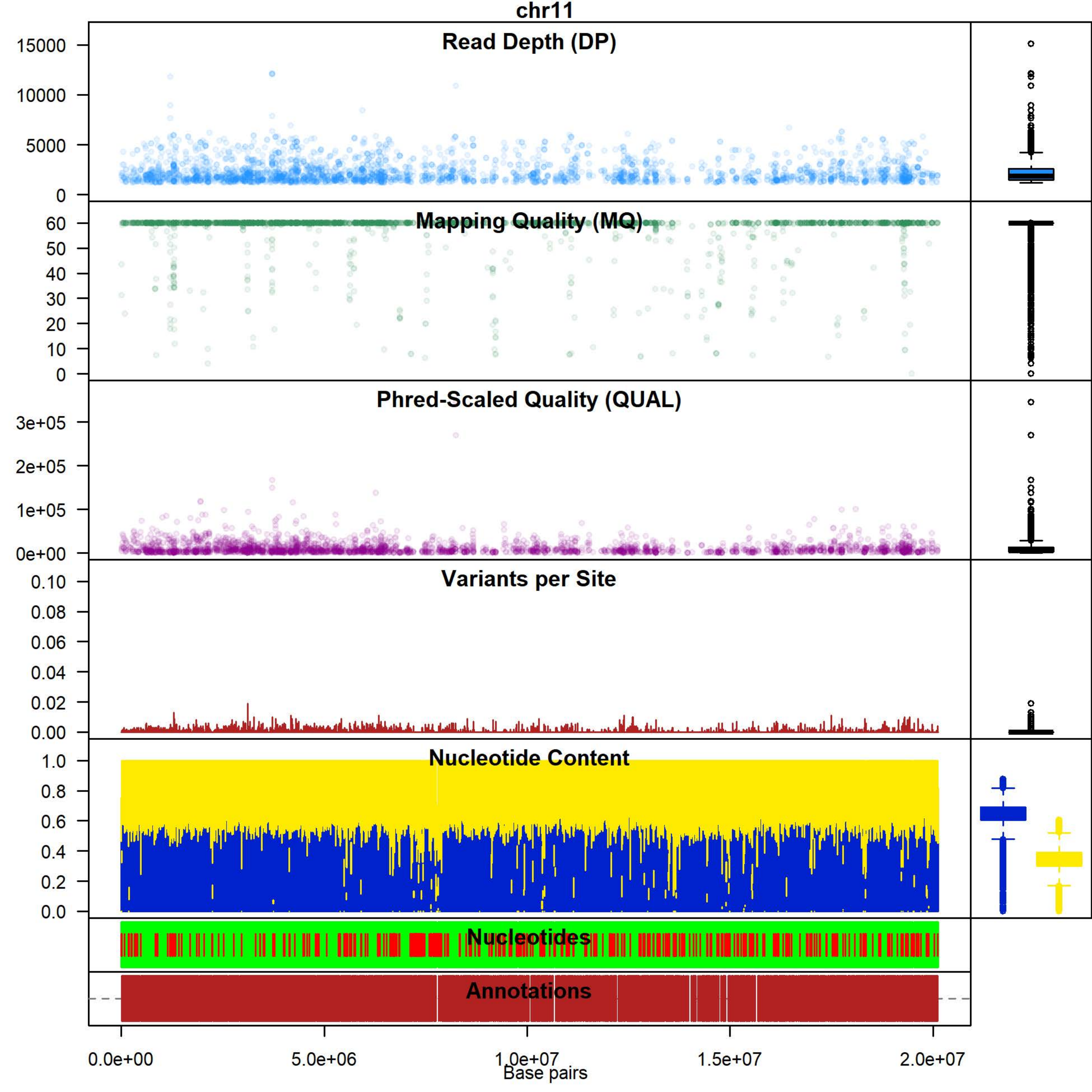

chr11
Read Depth (DP)
Mapping Quality (MQ)
Phred-Scaled Quality (QUAL)
Variants per Site
Nucleotide Content
Nucleotides
Annotations
Base pairs

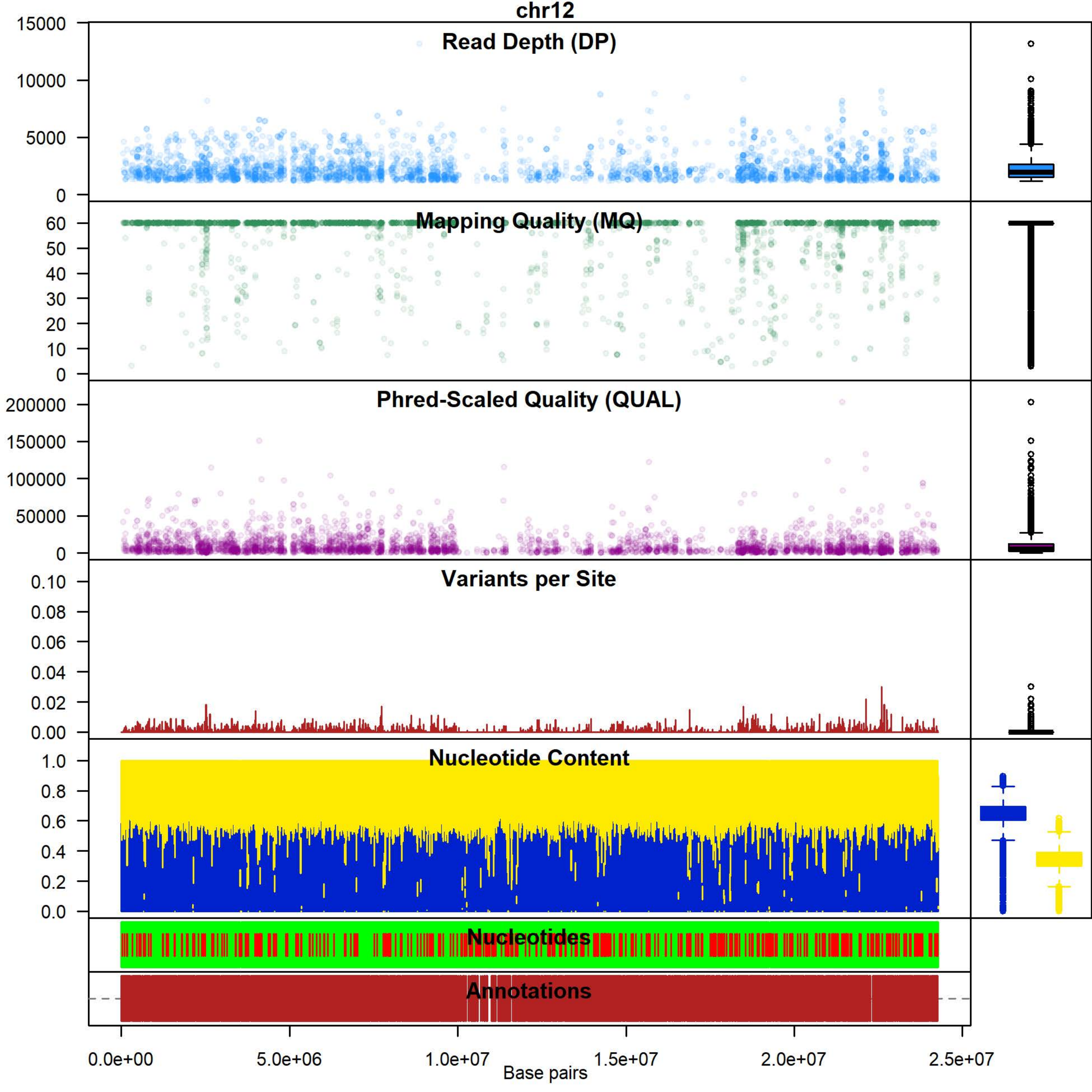
chr12
Read Depth (DP)
Mapping Quality (MQ)
Phred-Scaled Quality (QUAL)
Variants per Site
Nucleotide Content
Nucleotides
Annotations
Base pairs

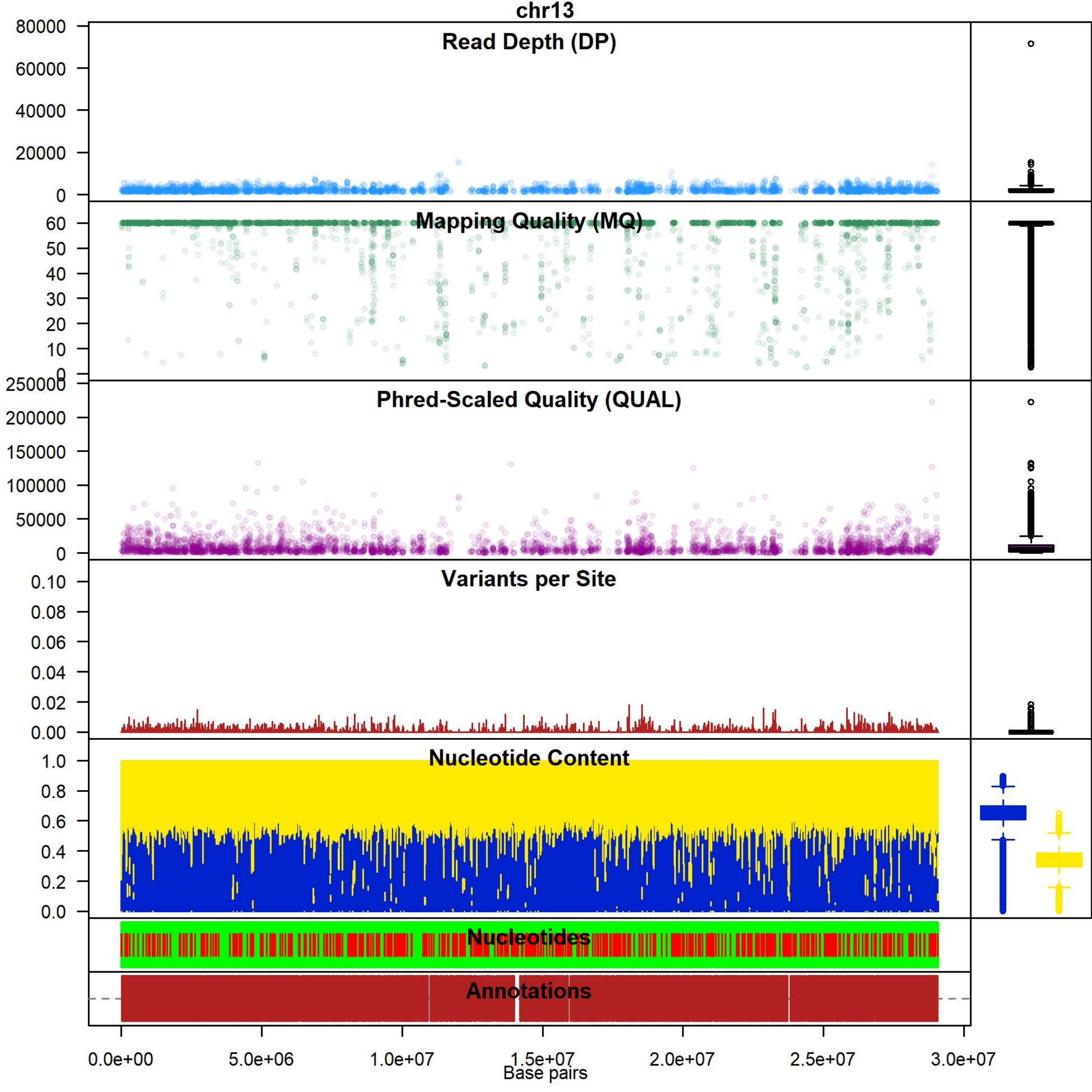

chr13
Read Depth (DP)
80000
60000
40000
20000
0
Mapping Quality (MQ)
60
50
40
30
20
10
0
Phred-Scaled Quality (QUAL)
250000
200000
150000
100000
50000
0
Variants per Site
0.10
0.08
0.06
0.04
0.02
0.00
Nucleotide Content
1.0
0.8
0.6
0.4
0.2
0.0
Nucleotides
Annotations
0.0e+00
5.0e+06
1.0e+07
1.5e+07
2.0e+07
2.5e+07
3.0e+07
Base pairs

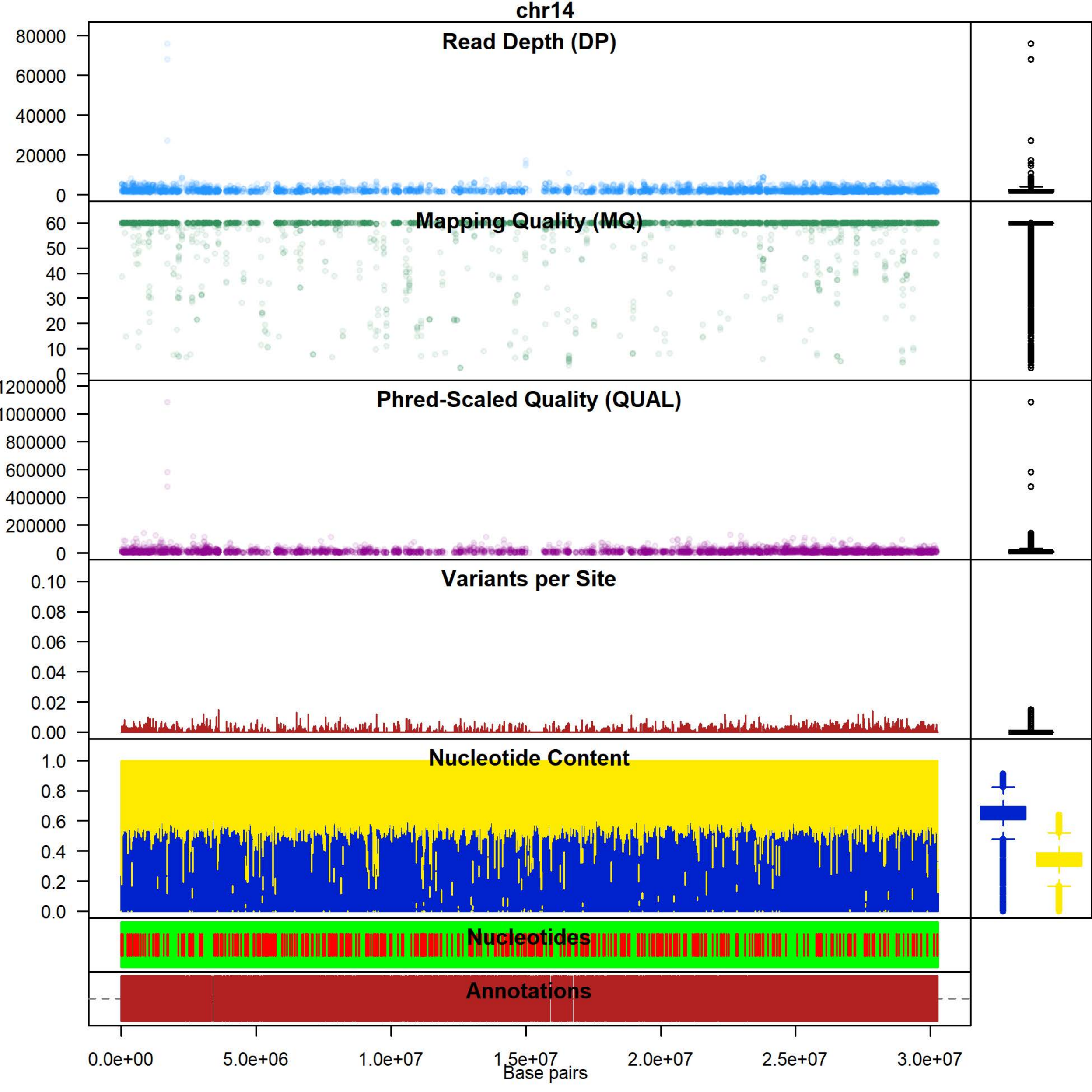

chr14
Read Depth (DP)
Mapping Quality (MQ)
Phred-Scaled Quality (QUAL)
Variants per Site
Nucleotide Content
Nucleotides
Annotations
Base pairs
0.0e+00
5.0e+06
1.0e+07
1.5e+07
2.0e+07
2.5e+07
3.0e+07

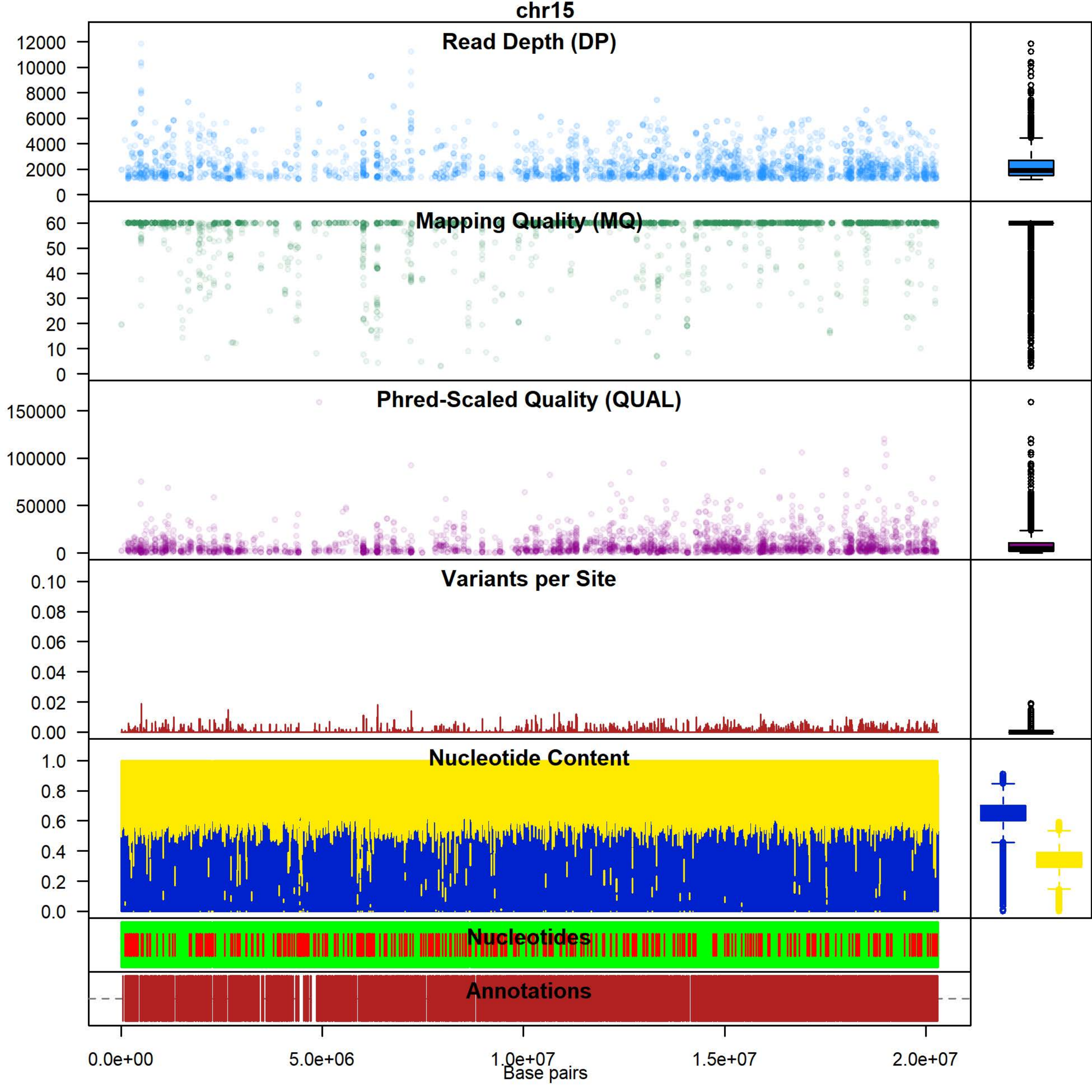

chr15
Read Depth (DP)
12000
10000
8000
6000
4000
2000
0
Mapping Quality (MQ)
60
50
40
30
20
10
0
Phred-Scaled Quality (QUAL)
150000
100000
50000
0
Variants per Site
0.10
0.08
0.06
0.04
0.02
0.00
Nucleotide Content
1.0
0.8
0.6
0.4
0.2
0.0
Nucleotides
Annotations
0.0e+00
5.0e+06
1.0e+07
1.5e+07
2.0e+07
Base pairs

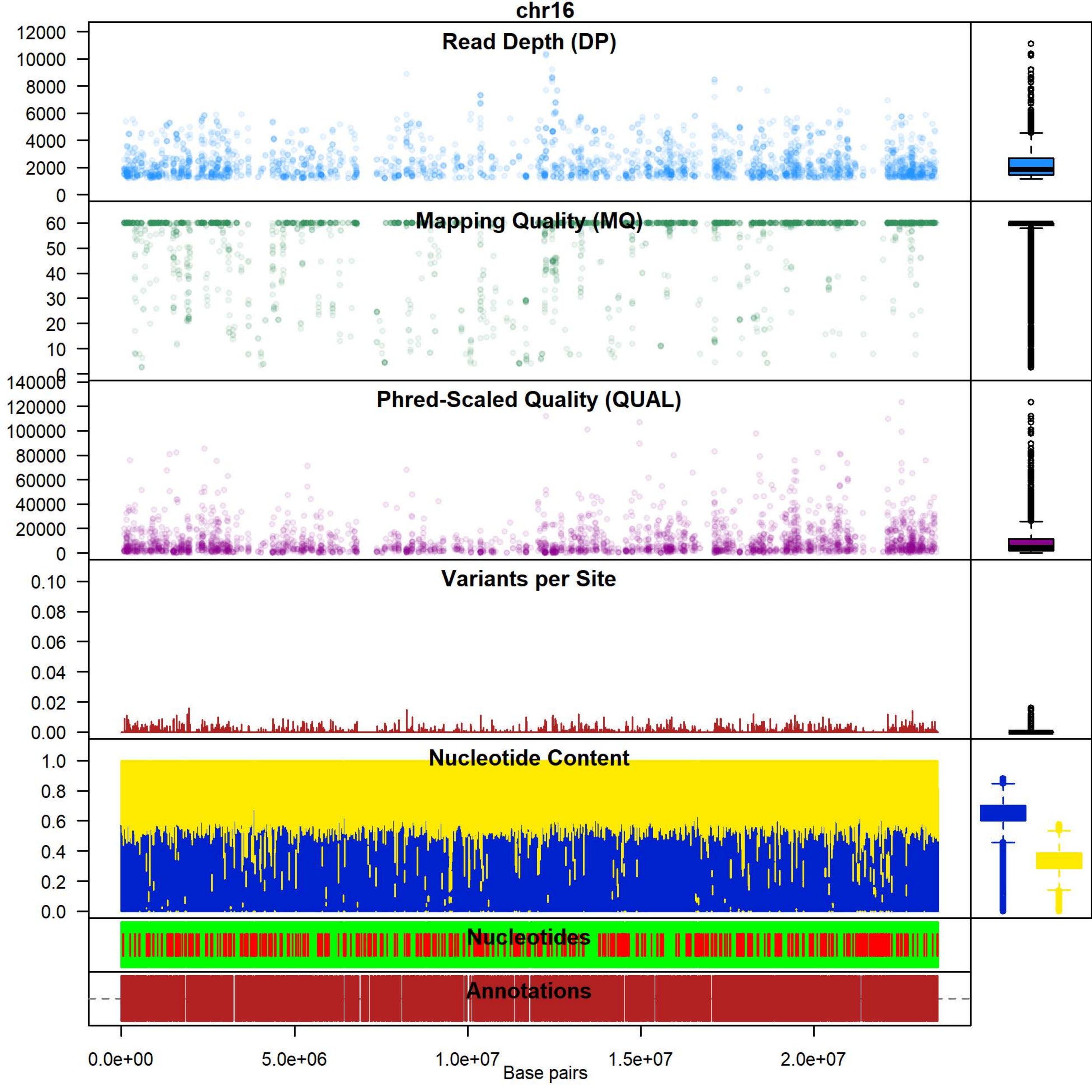

chr16
Read Depth (DP)
Mapping Quality (MQ)
Phred-Scaled Quality (QUAL)
Variants per Site
Nucleotide Content
Nucleotides
Annotations
0.0e+00
5.0e+06
1.0e+07
1.5e+07
2.0e+07
Base pairs

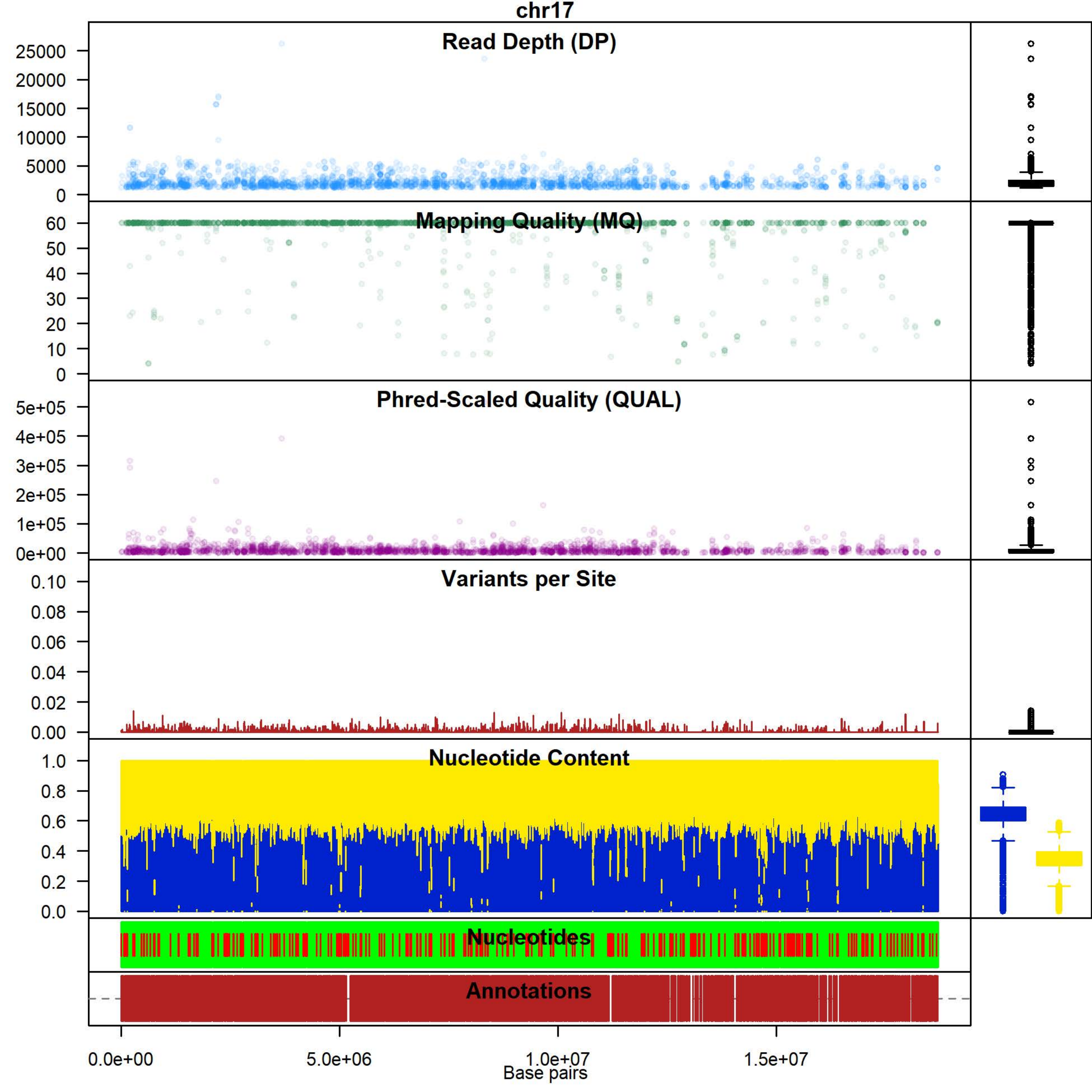
chr17
Read Depth (DP)
Mapping Quality (MQ)
Phred-Scaled Quality (QUAL)
Variants per Site
Nucleotide Content
Nucleotides
Annotations
Base pairs
0.0e+00
5.0e+06
1.0e+07
1.5e+07

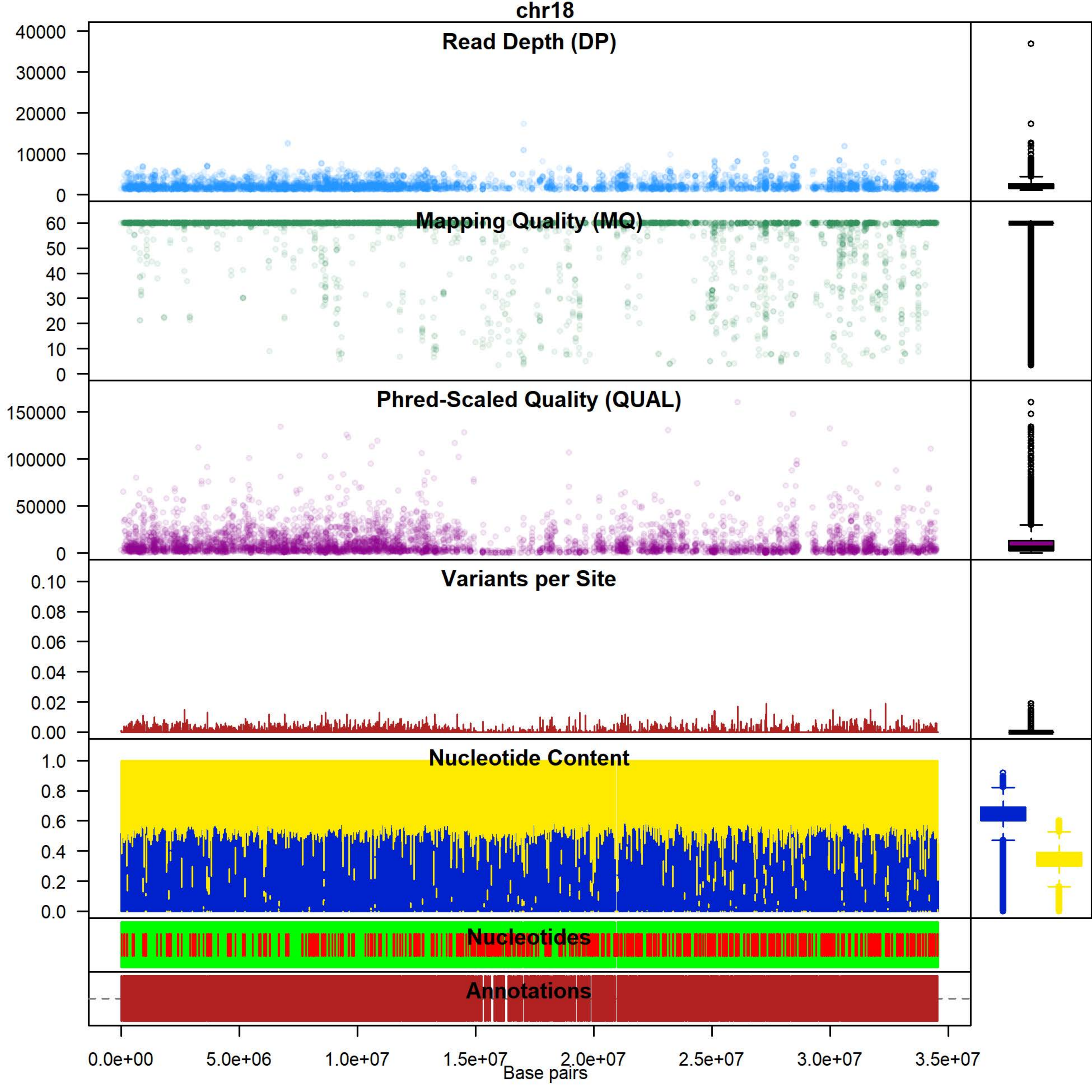

chr18
Read Depth (DP)
40000
30000
20000
10000
0
Mapping Quality (MQ)
60
50
40
30
20
10
0
Phred-Scaled Quality (QUAL)
150000
100000
50000
0
Variants per Site
0.10
0.08
0.06
0.04
0.02
0.00
Nucleotide Content
1.0
0.8
0.6
0.4
0.2
0.0
Nucleotides
Annotations
0.0e+00
5.0e+06
1.0e+07
1.5e+07
2.0e+07
2.5e+07
3.0e+07
3.5e+07
Base pairs

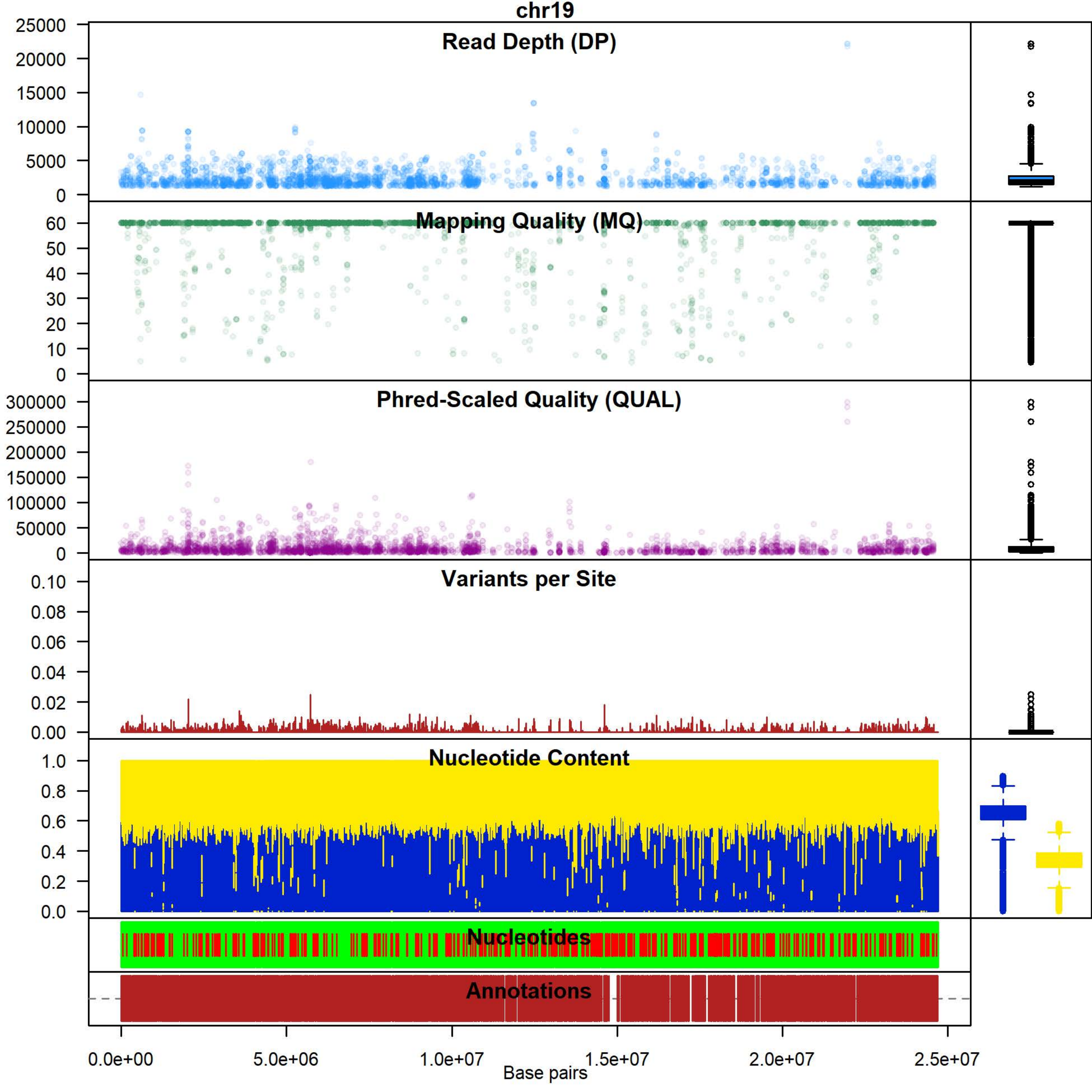
chr19
Read Depth (DP)
25000
20000
15000
10000
5000
0
Mapping Quality (MQ)
60
50
40
30
20
10
0
Phred-Scaled Quality (QUAL)
300000
250000
200000
150000
100000
50000
0
Variants per Site
0.10
0.08
0.06
0.04
0.02
0.00
Nucleotide Content
1.0
0.8
0.6
0.4
0.2
0.0
Nucleotides
Annotations
0.0e+00
5.0e+06
1.0e+07
1.5e+07
2.0e+07
2.5e+07
Base pairs
