# Supplementary material for: Characterization of Bunch Compactness in a Diverse Collection of Vitis vinifera L. Genotypes Enriched in Table Grape Cultivars Reveals New Candidate Genes Associated with Berry Number
Source: Plants (Basel). 2025 Apr 26;14(9):1308. doi: 10.3390/plants14091308 (PMC12073236; doi:10.3390/plants14091308)
Supplement: Supplementary file 1 [file plants-14-01308-s001.zip › Table S1.pdf]

Table S1 – Plant material used for the characterization of traits determining bunch compactness. A collection of cultivars with high genetic diversity due to their different provenances and reported utilization was considered for the study of traits involved in the compactness phenotype. There is a particular enrichment on modern table grape material which was not subjected to standard management practices regarding usage of plant growth regulator for thinning and berry enlargement since our focus was to observe the phenotype under a basal condition. Classification and provenance are those reported in the *Vitis* International Variety Catalogue (*V*IVC).

Prime name	VIVC code	GBS code	Population	Usage	Provenance
Agoumastos Kokkino	109	H35P16	Variety	Wine	Greece
Agourane	110	H48P11	Variety	Mixed	Algeria
Ak Uzyum Tagapskii	174	H49P11	Variety	Mixed	SUN (Formerly USSR)
Alburla	252	H42P27	Variety	Mixed	Ukraine
Alphonse Lavallee	349	H43P35	Variety	Mixed	France
Arbois Blanc	562	H41P6	Variety	Wine	France
Asyl Kara	731	H49P16	Variety	Mixed	Daghestan
Beauty Seedless	1065	H44P33	Variety	Table	United States of America
Blush Seedless	1472	H44P43	Variety	Table	United States of America
Burgrave De Hongrie	1901	H34P1	Variety	Table	Hungary
Cabernet Franc	1927	H41P71	Variety	Wine	France
Cabernet Sauvignon	1929	H35P13	Variety	Wine	France
Calmeria	2004	H43P66	Variety	Table	United States of America
Canner Seedless	2055	H47P6	Variety	Table	United States of America
Cardinal	2091	H47P11	Variety	Mixed	United States of America
Centennial Seedless	2380	H44P58	Variety	Table	United States of America
Cesar	2400	H35P7	Variety	Wine	France
Chilar	2558	H39P1	Variety	Wine	Armenia
Chouchillon	2619	H37P36	Variety	Wine	France
Dawn Seedless	3467	H42P40	Variety	Table	United States of America
Delizia Di Vaprio	3510	H42P6	Variety	Mixed	Italy
Dodrelyabi	3616	H42P42	Variety	Table	Georgia
Duc De Magenta	3703	H42P1	Variety	Table	France
Emperor	3904	H42P71	Variety	Mixed	United States of America
Exotic	4022	H43P10	Variety	Table	United States of America
Flame Seedless	4141	C1H1P3	Variety	Table	United States of America
Fue Fuki	4276	H47P21	Variety	Table	Japan
Galego Dourado	4325	H40P62	Variety	Wine	Portugal
Italia	5582	H43P74	Variety	Mixed	Italy
July Muscat	5855	H50P11	Variety	Mixed	United States of America
Kali Sahebi	5937	H49P31	Variety	Mixed	India
Korithi Aspro	6414	H50P1	Variety	Mixed	Greece
Kyoho	6597	H43P17	Variety	Table	Japan
Listan Prieto	6860	H36P26	Variety	Wine	Spain

Merlot Noir	7657	H41P61	Variety	Wine	France
Monastrell	7915	H35P56	Variety	Mixed	Spain
Muscat Fleur D'oranger	8221	H40P51	Variety	Mixed	France
Orlovi Nokti Beli	8807	H45P11	Variety	Table	Russian Federation
Csaba Gyongye	9166	H41P31	Variety	Mixed	Hungary
Perlette	9168	H42P21	Variety	Table	United States of America
Perlon	9170	H43P57	Variety	Mixed	Argentina
Pervenetz Praskoveisky	9195	H45P6	Variety	Mixed	Russian Federation
Pinot Noir	9279	H41P51	Variety	Wine	France
Plechistik	9558	H39P16	Variety	Wine	Russian Federation
Primitivo	9703	H41P36	Variety	Wine	Balkan
Queen	9841	H43P41	Variety	Table	United States of America
Retagliado Bianco	10035	H47P16	Variety	Mixed	Italy
Ruby Cabernet	10313	H41P46	Variety	Mixed	United States of America
Ruby Seedless	10314	H44P25	Variety	Table	United States of America
Sauvignon Gris	10792	H40P19	Variety	Wine	France
Rosa del Perú	1405	H43P25	Variety	Mixed	Peru
Schiava Grossa	10823	H40P76	Variety	Table	Italy
Silvaner Gruen	11805	H40P56	Variety	Wine	Austria
Sultanina	12051	H44P18	Variety	Table	Central Asia
Sugraone	12087	H44P66	Variety	Table	United States of America
Tinta Cao	12500	H37P17	Variety	Wine	Portugal
Gewurztraminer	12609	H40P21	Variety	Wine	Germany
Tsolikouri	12710	H38P11	Variety	Wine	Georgia
Verdot	12974	H41P16	Variety	Wine	France
Voskeat	13165	H49P1	Variety	Mixed	Armenia
Yai Izyum Rozovyi	13295	H35P26	Variety	Mixed	Daghestan
Yapincak	13311	H48P16	Variety	Mixed	Turkey
Autumn Seedless	13722	H47P26	Variety	Table	United States of America
Beitamouni	14790	H32P6	Variety	Table	Lebanon
Malahy	15013	H33P1	Variety	Table	Iran
Mehdik	15029	H45P26	Variety	Table	Iran
Red Seedless	15452	H40P20	Variety	Table	Argentina
Crimson Seedless	16019	H44P49	Variety	Table	United States of America
Chirai Obak	16251	H32P23	Variety	Wine	SUN (Formerly USSR)
Tsitsa Kaprei	16449	H45P17	Variety	Mixed	Moldova
Sultanine Monococco	17508	H45P21	Variety	Table	Turkey
Autumn Royal	17651	C1H1P11	Variety	Table	United States of America
Plant Du Maroc E	24372	H45P1	Variety	Table	Morocco
Espadeiro Tinto	24552	H40P72	Variety	Wine	Portugal
Medawar	24925	H32P17	Variety	Wine	Israel

Bezzoul Kelba El Bidha Gabes	24997	H42P16	Variety	Mixed	Tunisia
Kapistoni Tetri	25033	H40P66	Variety	Wine	Georgia
Moscatel Alba Rosa Inia	25147	H42P11	Variety	Table	Chile
Patagonia	26277	H44P73	Variety	Table	Argentina
Moscatel De Oeiras Faux	42107	H47P1	Variety	Mixed	Portugal
Selección_2	N.R.	C1H5P5	Variety	Table	Chile
Selección_2047	N.R.	C1H4P3	Variety	Table	Chile
Selección_23	N.R.	C1H6P2	Variety	Table	Chile
Selección_6_14_205	N.R.	C1H5P8	Variety	Table	Chile
Local selection	N.R.	H43P49	Variety	Table	Chile
C406E9H1P166	N.R.	C406E9H1P166	Segregating line	Table	Chile
C406E9H1P216	N.R.	C406E9H1P216	Segregating line	Table	Chile
C5E9H1P1	N.R.	C5E9H1P1	Segregating line	Table	Chile
C5E9H1P2	N.R.	C5E9H1P2	Segregating line	Table	Chile
C5E9H1P31	N.R.	C5E9H1P31	Segregating line	Table	Chile
C5E9H1P38	N.R.	C5E9H1P38	Segregating line	Table	Chile
C895E9H3P220	N.R.	C895E9H3P220	Segregating line	Table	Chile
C895E9H3P225	N.R.	C895E9H3P225	Segregating line	Table	Chile
C895E9H3P228	N.R.	C895E9H3P228	Segregating line	Table	Chile
C895E9H3P229	N.R.	C895E9H3P229	Segregating line	Table	Chile
C940E9H4P27	N.R.	C940E9H4P27	Segregating line	Table	Chile
C940E9H4P33	N.R.	C940E9H4P33	Segregating line	Table	Chile
C940E9H4P43	N.R.	C940E9H4P43	Segregating line	Table	Chile
C940E9H4P5	N.R.	C940E9H4P5	Segregating line	Table	Chile
C940E9H4P7	N.R.	C940E9H4P7	Segregating line	Table	Chile
C990E11H2P16	N.R.	C990E11H2P16	Segregating line	Table	Chile
C990E11H2P164	N.R.	C990E11H2P164	Segregating line	Table	Chile
C990E11H2P17	N.R.	C990E11H2P17	Segregating line	Table	Chile
C990E11H2P188	N.R.	C990E11H2P188	Segregating line	Table	Chile
C990E11H2P22	N.R.	C990E11H2P22	Segregating line	Table	Chile
C990E11H2P35	N.R.	C990E11H2P35	Segregating line	Table	Chile
C990E11H2P83	N.R.	C990E11H2P83	Segregating line	Table	Chile

C990E11H2P84	N.R.	C990E11H2P84	Segregating line	Table	Chile
C992E11H6P113	N.R.	C992E11H6P113	Segregating line	Table	Chile
C992E11H6P16	N.R.	C992E11H6P16	Segregating line	Table	Chile
C992E11H7P29	N.R.	C992E11H7P29	Segregating line	Table	Chile
C992E11H7P32	N.R.	C992E11H7P32	Segregating line	Table	Chile
C992E11H7P38	N.R.	C992E11H7P38	Segregating line	Table	Chile
C992E11H7P45	N.R.	C992E11H7P45	Segregating line	Table	Chile
C992E11H7P46	N.R.	C992E11H7P46	Segregating line	Table	Chile

*N.R. Not Registered
