# Supplementary material for: Characterization of Bunch Compactness in a Diverse Collection of Vitis vinifera L. Genotypes Enriched in Table Grape Cultivars Reveals New Candidate Genes Associated with Berry Number
Source: Plants (Basel). 2025 Apr 26;14(9):1308. doi: 10.3390/plants14091308 (PMC12073236; doi:10.3390/plants14091308)
Supplement: Supplementary file 1 [file plants-14-01308-s001.zip › Table S2.pdf]

Table S2 – Descriptive statistics for quantitative indexes describing BC. The compactness indexes proposed by Tello & Ibañez (2015) were calculated for our dataset across two seasons. Descriptive statistics are shown for a total of 768 and 622 observations for the first and second seasons, respectively.

Trait	Season	Mean	Standard deviation	Minimum	1st Quartile	Median	3rd Quartile	Maximum
CI-01	2022	4.682	3.164	0.1739	2.641	3.929	5.84	22.60
	2023	4.639	2.553	0.6597	2.897	4.119	5.82	18.38
CI-02	2022	2.095	1.318	0.2809	1.172	1.832	2.66	13.61
	2023	2.009	1.114	0.3476	1.235	1.808	2.51	9.61
CI-03	2022	8.660	4.443	0.8152	5.306	8.128	10.80	32.03
	2023	8.636	4.330	1.7766	5.667	7.946	10.78	33.57
CI-06	2022	2.952	2.185	0.1132	1.700	2.485	3.60	18.52
	2023	2.800	1.487	0.5024	1.767	2.473	3.50	12.69
CI-08	2022	68.084	59.500	2.0378	32.132	53.419	85.13	611.10
	2023	69.105	46.224	5.9209	37.202	57.016	89.82	333.01
CI-10	2022	18.976	9.235	1.0047	12.627	17.860	24.42	54.16
	2023	20.131	10.483	2.7518	13.230	18.440	24.39	94.36
CI-12	2022	1.025	0.611	0.0429	0.611	0.898	1.30	5.62
	2023	0.975	0.651	0.1004	0.578	0.842	1.22	6.74
CI-14	2022	1.321	0.856	0.1994	0.721	1.142	1.64	6.87
	2023	1.214	0.647	0.2170	0.760	1.093	1.49	5.27
CI-16	2022	-2.355	0.697	-7.4875	-2.739	-2.220	-1.86	-1.10
	2023	-2.199	0.726	-8.0592	-2.533	-2.078	-1.73	-0.93
CI-17	2022	3.815	7.059	0.0415	0.958	2.178	4.18	137.10
	2023	3.004	3.511	0.0765	0.960	1.928	3.56	31.17
CI-18	2022	1.698	3.239	0.0119	0.329	0.809	1.81	54.93
	2023	1.116	1.673	0.0185	0.261	0.591	1.33	16.61
CI-19	2022	0.519	0.876	0.0042	0.119	0.253	0.55	9.97
	2023	0.334	0.475	0.0053	0.072	0.177	0.39	3.88
