# Supplementary material for: Characterization of Bunch Compactness in a Diverse Collection of Vitis vinifera L. Genotypes Enriched in Table Grape Cultivars Reveals New Candidate Genes Associated with Berry Number
Source: Plants (Basel). 2025 Apr 26;14(9):1308. doi: 10.3390/plants14091308 (PMC12073236; doi:10.3390/plants14091308)
Supplement: Supplementary file 1 [file plants-14-01308-s001.zip › Table S4.pdf]

Table S4 – Summary of the predicted effects of the alternative alleles on the closest annotated genes. The predicted effect based on the alternative allele is reported. Categories are those reported by Snpeff (Cingolani et al., 2012). Some sites could have multiple predicted effects but only one of them was considered

Season	Predicted effect	Number of cases
S1	3_prime_UTR_variant	16
S2	3_prime_UTR_variant	19
S1	5_prime_UTR_variant	2
S2	5_prime_UTR_variant	9
S1	downstream_gene_variant	51
S2	downstream_gene_variant	50
S1	intergenic_region	8
S2	intergenic_region	14
S1	intron_variant	60
S2	intron_variant	68
S1	missense_variant	39
S2	missense_variant	71
S1	missense_variant&splice_region_variant	1
S2	missense_variant&splice_region_variant	2
S1	splice_region_variant&intron_variant	3
S2	splice_region_variant&intron_variant	3
S1	stop_gained	2
S2	stop_gained	3
S1	stop_lost	1
S1	stop_retained_variant	1
S2	stop_retained_variant	1
S1	synonymous_variant	43
S2	synonymous_variant	30
S1	upstream_gene_variant	56
S2	upstream_gene_variant	86
