# Supplementary figures and images for: Characterization of Bunch Compactness in a Diverse Collection of Vitis vinifera L. Genotypes Enriched in Table Grape Cultivars Reveals New Candidate Genes Associated with Berry Number
Source: Plants (Basel). 2025 Apr 26;14(9):1308. doi: 10.3390/plants14091308 (PMC12073236; doi:10.3390/plants14091308)

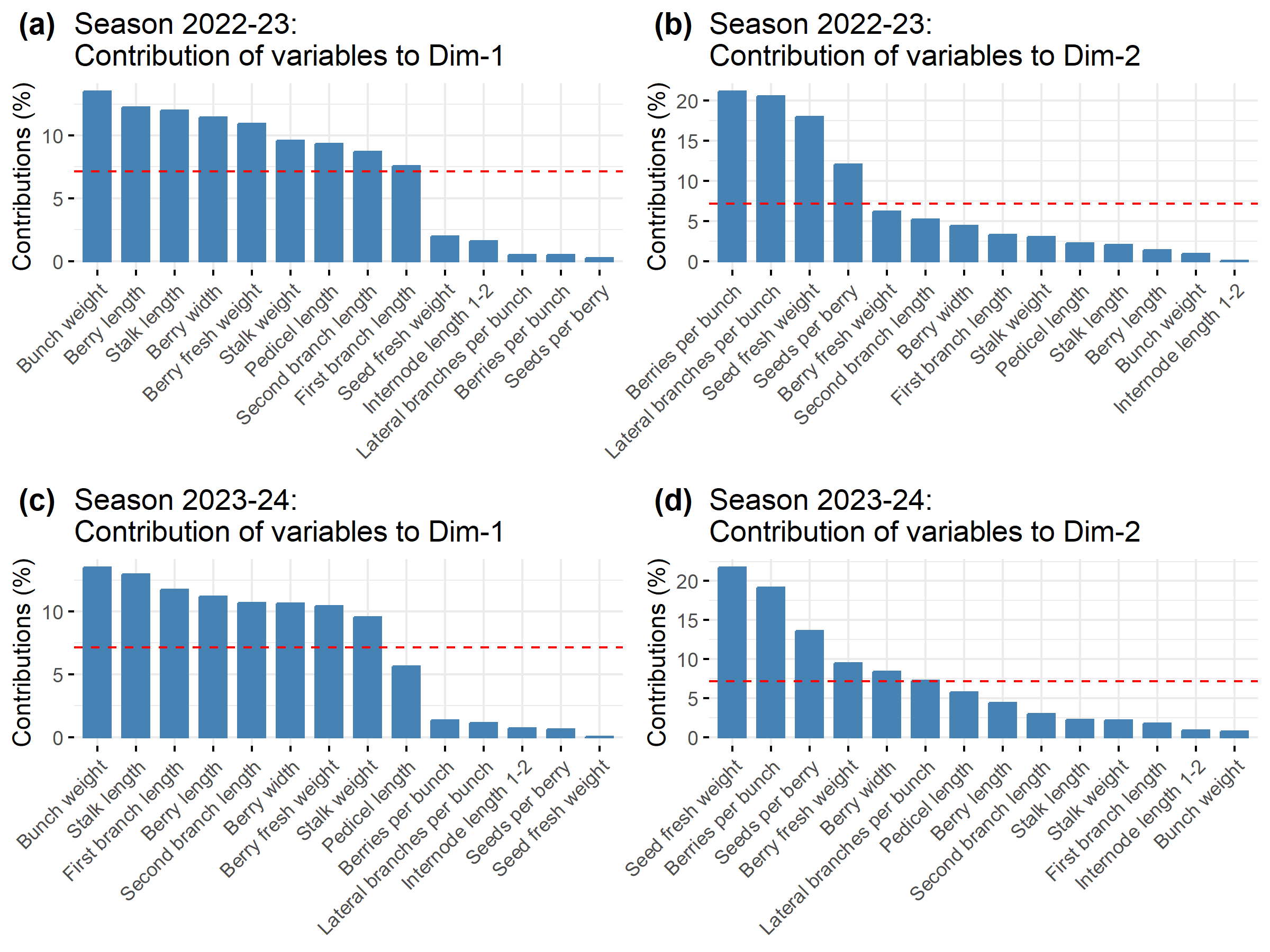

Supplement: Supplementary file 1 [file plants-14-01308-s001.zip › Figure S1 - Contributions charts - Traits to PCA 2025_03_27.png]

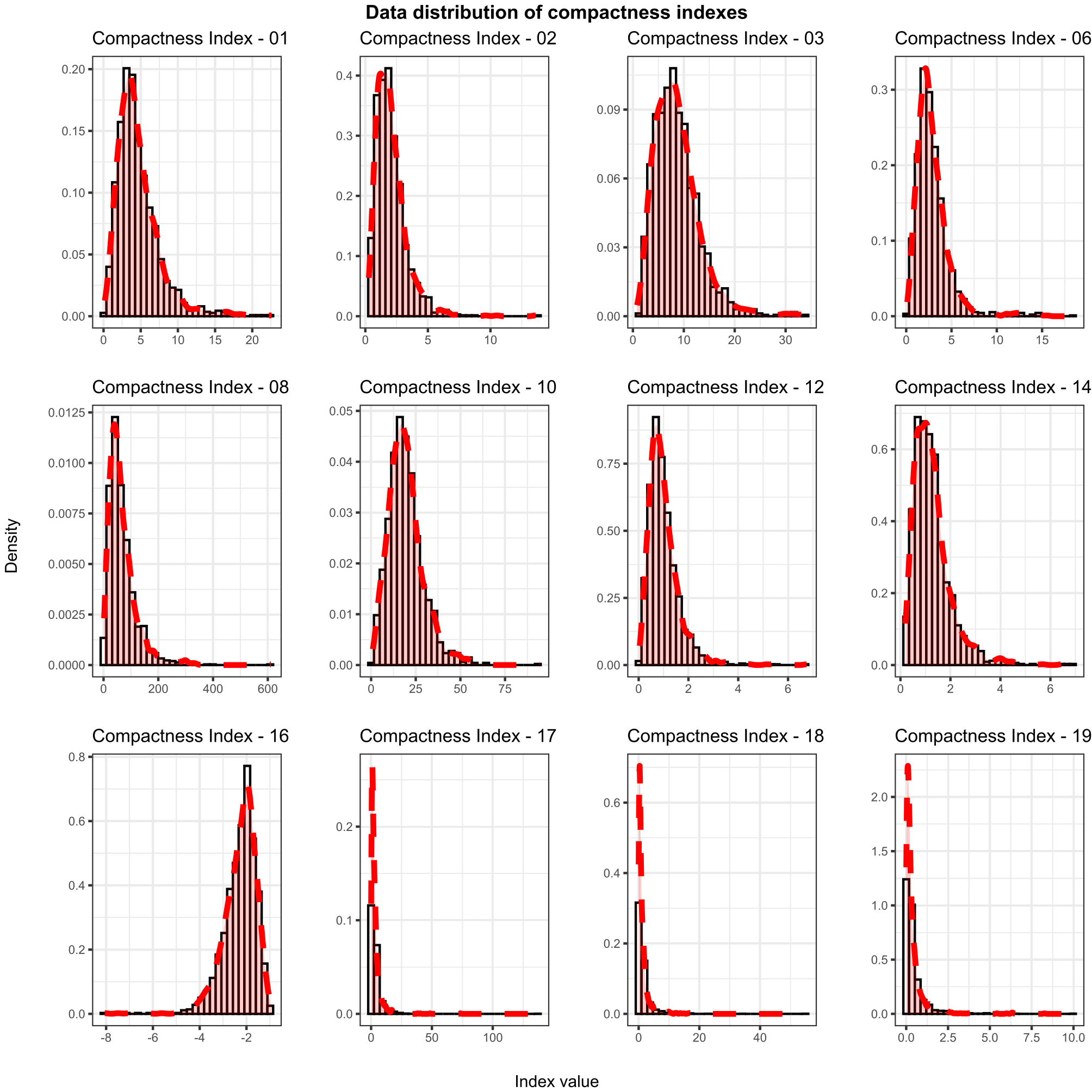

Supplement: Supplementary file 1 [file plants-14-01308-s001.zip › Figure S2 - Compactness indexes distribution.png]

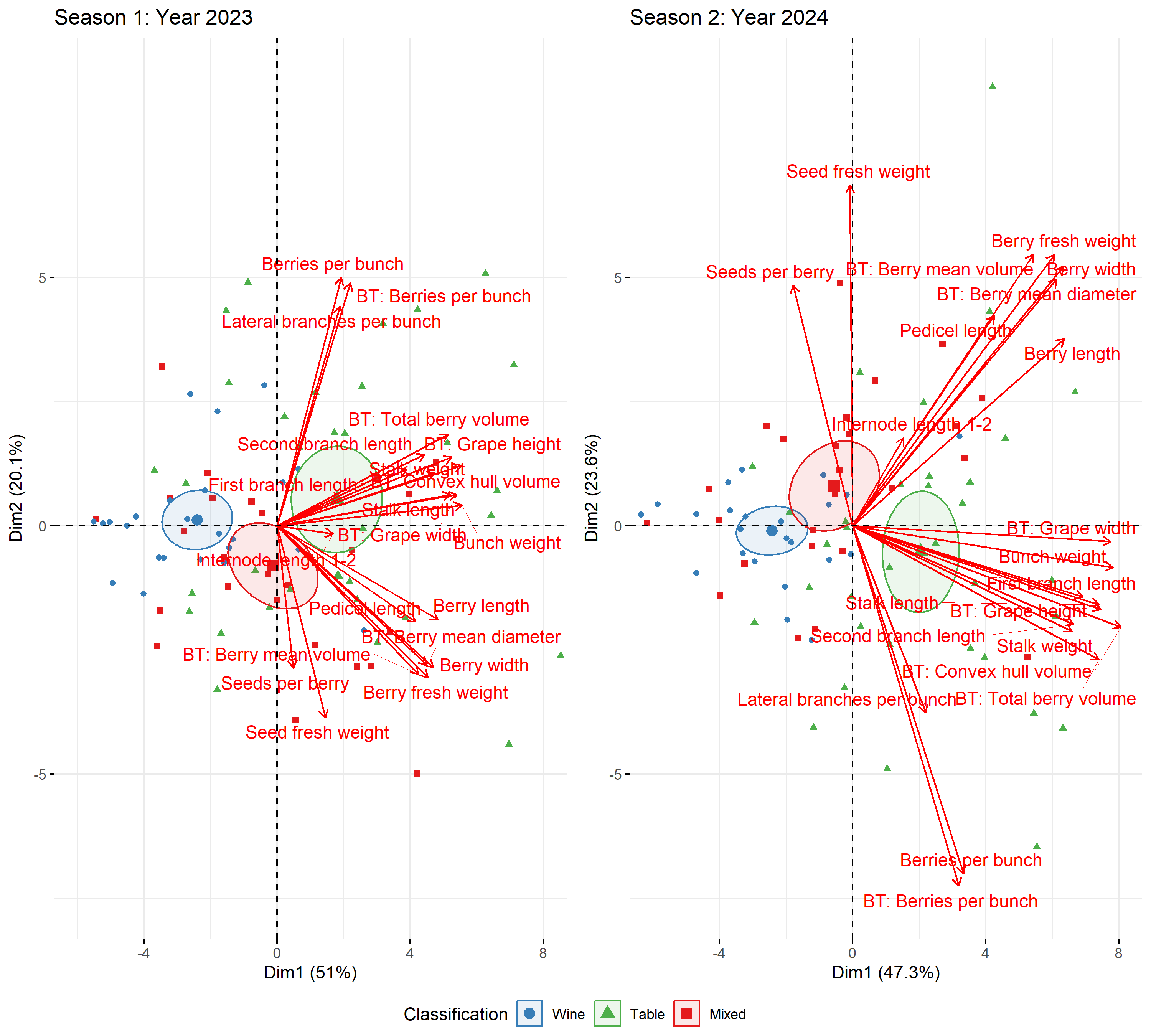

Supplement: Supplementary file 1 [file plants-14-01308-s001.zip › Figure S3 - Biplot both seasons - MM and BT traits.png]

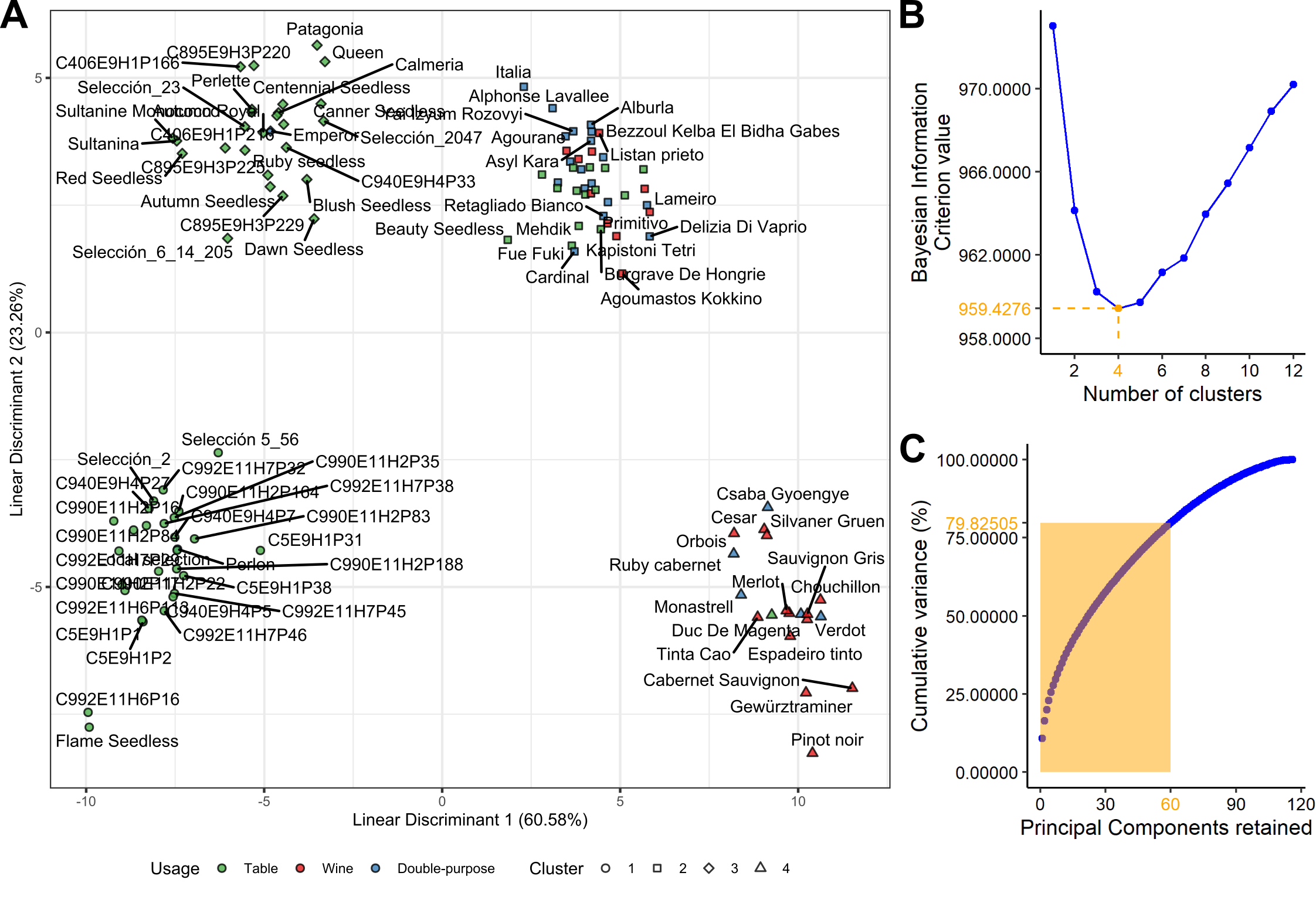

Supplement: Supplementary file 1 [file plants-14-01308-s001.zip › Figure S4 - Structure population.png]

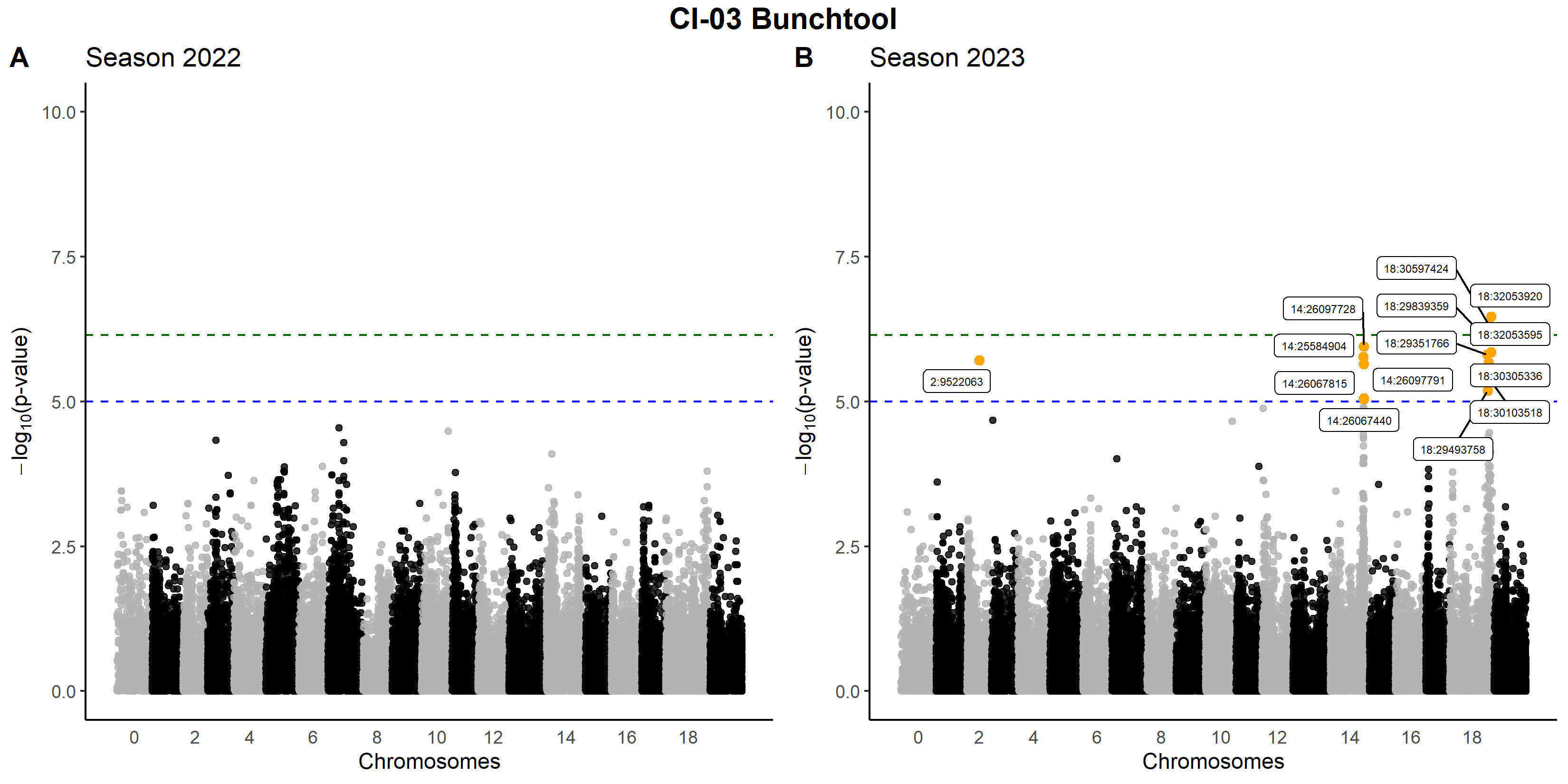

Supplement: Supplementary file 1 [file plants-14-01308-s001.zip › Figure S5 - CI_03_BT 2025_02_18.png]

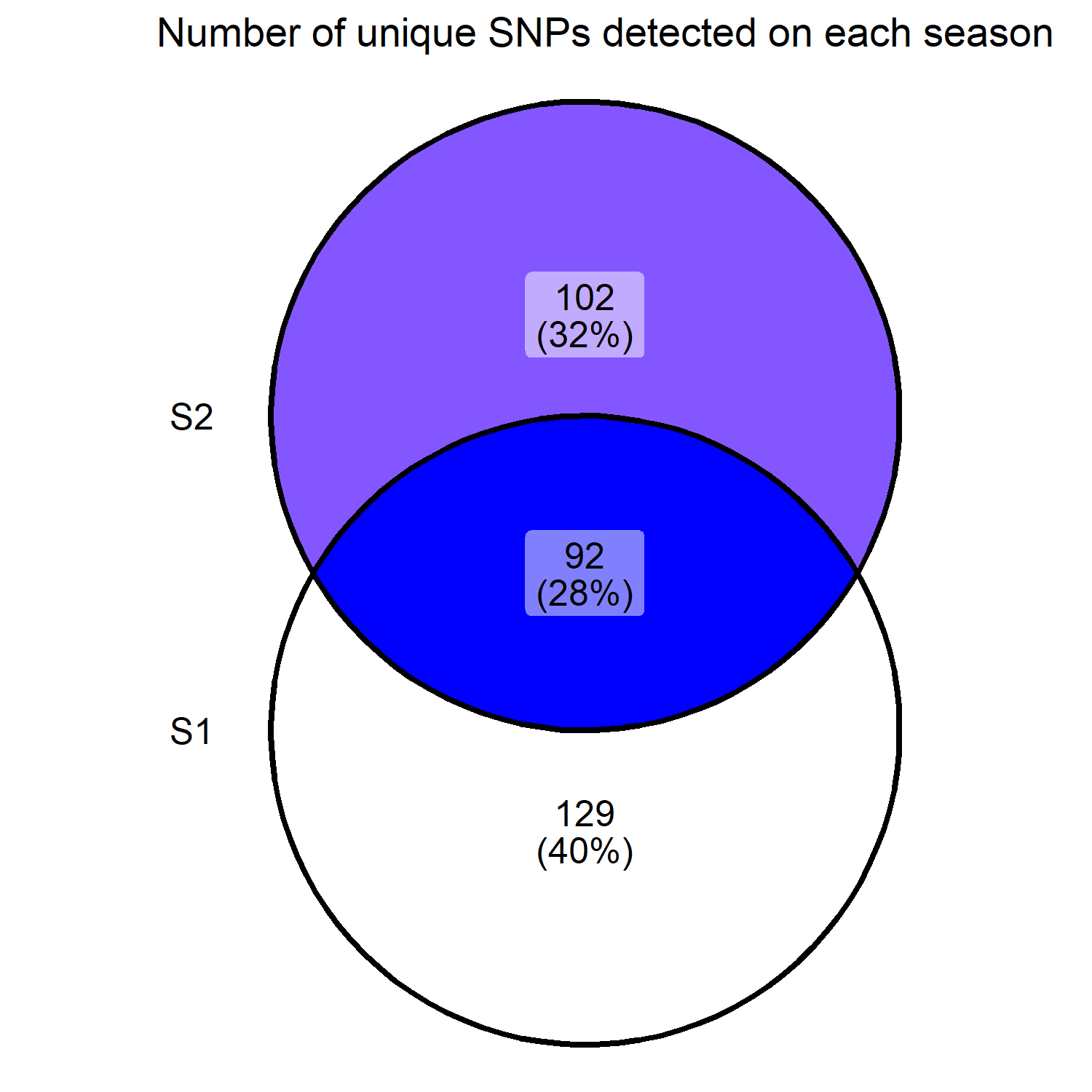

Supplement: Supplementary file 1 [file plants-14-01308-s001.zip › Figure S6 - Venn diagram unique SNPs.png]

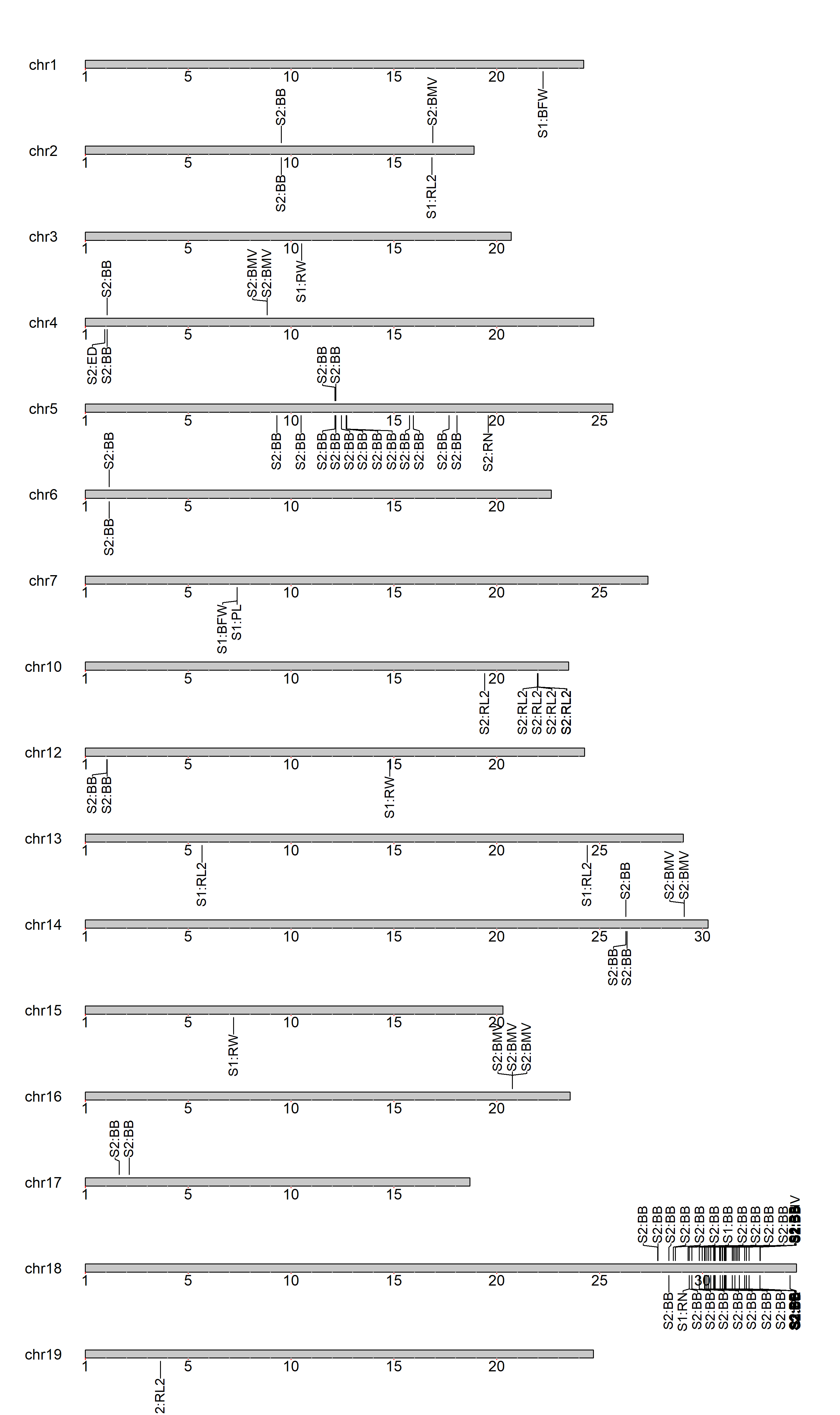

Supplement: Supplementary file 1 [file plants-14-01308-s001.zip › Figure S7 - Idiogram significant loci.png]

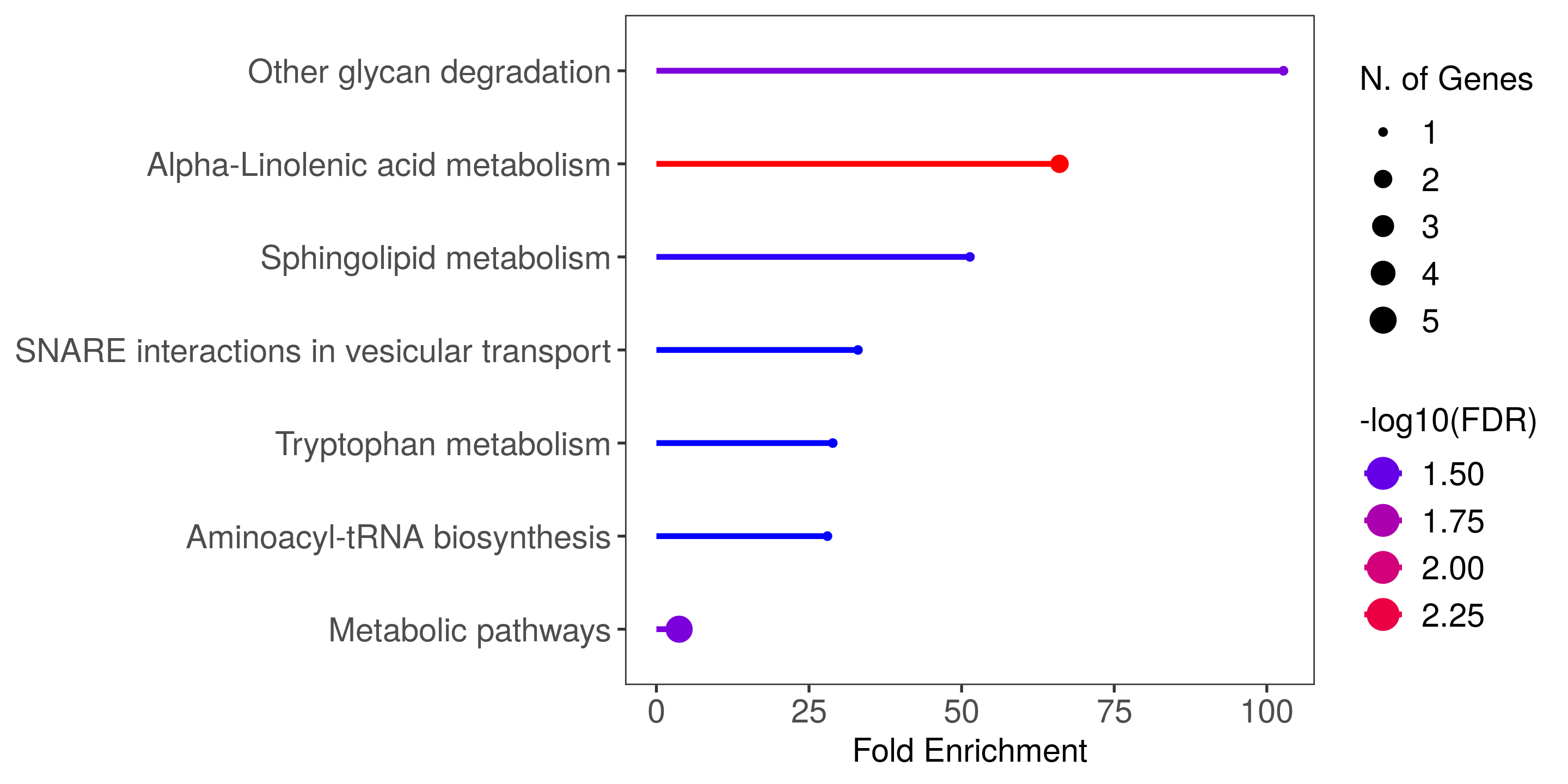

Supplement: Supplementary file 1 [file plants-14-01308-s001.zip › Figure S8 - Dotplot enrichment analysis.png]
